# Substance P–NK1R signaling of keratinocytes initiates the skin inflammatory environment required for allergic contact dermatitis

**DOI:** 10.1172/jci.insight.205826

**Published:** 2026-09-01

**Authors:** Sumeet Manandhar, Mohna Bandyopadhyay, Olga A. Tkacheva, William J. Shufesky, Gregory A. Gibson, Simon C. Watkins, Adrian E. Morelli, Adriana T. Larregina

**Affiliations:** 1 Department of Dermatology,; 2 Thomas E. Starzl Transplantation Institute,; 3 Department of Surgery,; 4 Department of Cell Biology and Center for Biological Imaging, and; 5 Department of Immunology, University of Pittsburgh, School of Medicine, Pittsburgh, Pennsylvania, USA.; 6 McGowan Center for Regenerative Medicine, University of Pittsburgh, Pittsburgh, Pennsylvania, USA.

**Keywords:** Dermatology, Immunology, G protein-coupled receptors, Innate immunity, Skin

## Abstract

Allergic contact dermatitis (ACD), a recurrent inflammatory skin disorder, affects 21% of humans and is the second leading cause of occupational diseases in the United States. ACD is initiated by the innate immune response to skin-contact sensitizers potentiated by the neuropeptide substance P (SP). Skin sensitizers stimulate SP-secreting sensory nerves and trigger proinflammatory functions of keratinocytes expressing the neurokinin 1 receptor (NK1R). Nevertheless, the neuroimmune regulation of hapten-initiated skin inflammation remains incompletely elucidated. Using *K14^Cre/+^ NK1R^KO^* mice skin-sensitized with 2,4-dinitrochlorobenzene (DNCB), we demonstrate that NK1R deletion exclusively in keratinocytes prevents hapten-initiated skin inflammation, impairs the mobilization of conventional dendritic cells to draining lymph nodes, and blocks the elicitation of the contact hypersensitivity reaction to the same extent observed in global *Tac1^KO^* (without SP) and *NK1R^KO^* mice. The DNCB effects were restored by skin co-administration of IL-1β and TNF-α. SP-NK1R signaling of mouse and human keratinocytes increased transcripts encoding proteins of the NLRP3 inflammasome. Although DNCB and SP induced pro–IL-1β synthesis, only SP triggered intracellular Ca^2+^ increase, NFATc1 nuclear translocation, and synthesis of TNF-α, a cytokine mediating systemic inflammation in ACD. Our data identifying SP-NK1R signaling of keratinocytes as a key mechanism for ACD provide relevant insight for therapies targeting skin neuroimmune interactions.

## Graphical abstract

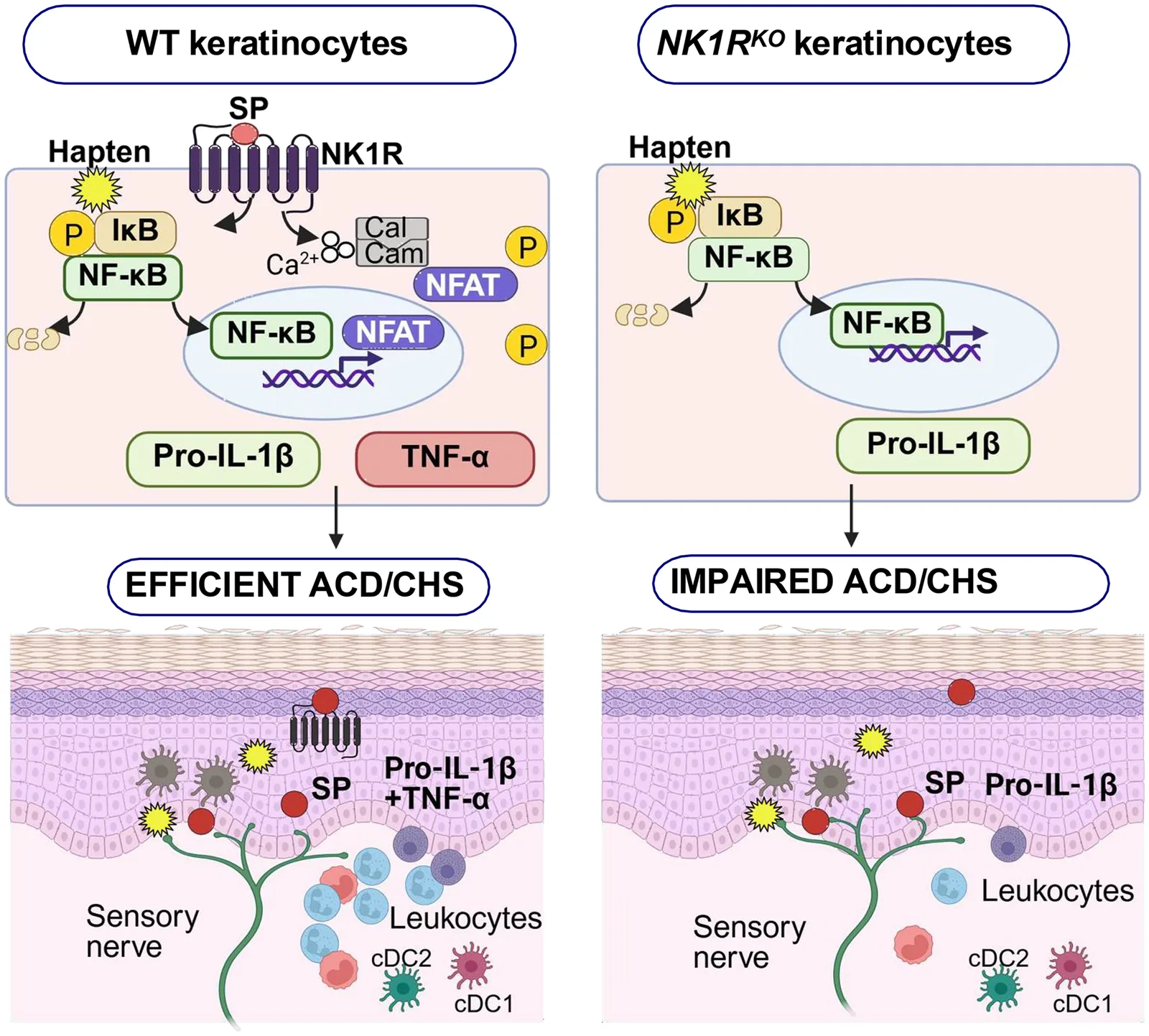

## Introduction

Allergic contact dermatitis (ACD) is a severe recurrent inflammatory skin disorder that affects up to 21% of the human population and is the second leading cause of occupational diseases in the United States (1–3). Common interventions for ACD include avoiding exposure to contact sensitizers, and administration of corticosteroids that, if given systemically, can cause generalized immunosuppression (1, 3). Hence, prevention and treatment of ACD would greatly benefit from cell type– and antigen-specific therapies. Nevertheless, gaps in our knowledge of the molecular mechanisms that induce ACD preclude development of specific treatments for the disease (1, 3).

Stimulation of skin sensory nerve endings by haptens induces the release of the proinflammatory neuropeptide substance P (SP), which amplifies the inflammatory response initiated by exposure to contact sensitizers (4–8). This cutaneous inflammatory environment, triggered by local secretion of IL-1β and TNF-α, is required to activate the immunostimulatory and migratory function of skin-resident conventional dendritic cells (cDCs). These immunostimulatory conventional DCs transport haptenated peptides to skin-draining lymph nodes (sdLNs), where they prime hapten-specific T cells, accounting for the sensitization phase of ACD. Following re-exposure to the contact sensitizer, hapten-specific T cells account for the effector phase and the recurrent flare-ups of the disease (9–14). Accordingly, the innate and adaptive immune responses stimulated by skin exposure to contact sensitizers are impaired in the absence of sensory nerve fibers, SP, or the SP-high-affinity neurokinin 1 receptor (NK1R) (4–6, 15–18).

Keratinocytes, the most abundant cells of the epidermis, acquire a potent proinflammatory function following exposure to contact sensitizers (19–24). Hence, several laboratories use in vitro models of mouse and human keratinocytes to test the inflammatory potency of contact sensitizers (22, 25–30). Mechanistically, the cutaneous inflammation triggered by extreme chemical haptens like 2,4-dinitrochlorobenzene (DNCB), fluorobenzene, and trinitro-chlorobenzene (TNCB), as well as by metal contact sensitizers like NiSO_4_, depends at least in part on activation of the NLRP3 inflammasome in keratinocytes resulting in synthesis of pro–IL-1β and pro–IL-18 and secretion of IL-1β and IL-18, respectively (31–37). Simultaneously, the irritant effect of DNCB triggers the secretion of SP by peptidergic neuronal fibers that extend to the epidermis (38–45). Interestingly, keratinocytes constitutively express the NK1R and undergo proinflammatory changes in response to SP (18, 46–49). In this regard, our group demonstrated that skin co-administration of haptens and NK1R antagonists impairs the development of contact hypersensitivity (CHS) and deletion of NK1R in keratinocytes abrogates the CHS response to DNCB (18). These results strongly suggest that keratinocytes are early targets of SP released locally and that SP-NK1R signaling in keratinocytes participates in the initiation of the skin innate immune response, leading to sensitization of ACD. Nevertheless, the molecular mechanisms that mediate the proinflammatory effects of the SP-NK1R axis in keratinocytes exposed to contact sensitizers, which could be targets of specific immunotherapies for ACD, remain to be elucidated.

Here we investigated the intracellular pathways signaled by the SP-NK1R axis following exposure of keratinocytes to a contact sensitizer. We tested the effects of DNCB because it (a) is a strong contact sensitizer used broadly to analyze the pathophysiology of CHS, (b) sensitizes humans, and (c) requires the SP-NK1R axis to induce ACD (4–6, 18, 31, 40). Our results demonstrate that SP-NK1R signaling of human and mouse keratinocytes cultured in vitro increases the expression of transcripts encoding NLRP3 inflammasome subunits and mRNA and protein synthesis of pro–IL-1β and TNF-α. In the same in vitro models, DNCB augmented NK1R expression and synthesis of pro–IL-1β in keratinocytes but failed to induce TNF-α. Consequently, DNCB applied topically to the skin of *K14^Cre/+^ NK1R^KO^* mice did not induce efficient sensitization to the hapten, resulting in impaired elicitation of the CHS response, effects that were restored by local co-administration of IL-1β and TNF-α during skin exposure to the hapten.

## Results

### The SP-NK1R axis is required for the skin innate immunity triggered by DNCB and croton oil.

Signaling of the NK1R by SP potentiates the adaptive immune response of the CHS, the mouse equivalent of ACD, to strong contact sensitizers (6, 8, 18). However, the roles of SP or the NK1R at early stages of the CHS sensitization are not completely understood. Therefore, we tested whether the SP-NK1R axis is required for the skin innate immunity underlying the irritant effect of the contact sensitizer. This innate immune response is necessary for the efficient development of the adaptive immune response that accounts for the effector phase of the CHS (1, 3, 12, 18). To address the role of SP in the skin irritant effect of haptens, *Tac1^KO^* (lacking SP) or WT mice were sensitized on the right ear with topical application of DNCB (1% in 1:4 acetone/olive oil) or treated on the left ear with vehicle. For this experiment, the sensitization in the mouse ear was chosen because it allows monitoring of the innate immune response by measurement of the increase of ear thickness following application of the contact sensitizer, which cannot be done in mice sensitized in the abdominal skin. The increase of ear thickness was assessed 24, 48, and 72 hours after sensitization, and parameters indicative of local innate immunity were analyzed in tissue samples of ears at the peak of the inflammatory response to DNCB, 24 hours after sensitization (Supplemental Figure 1A; supplemental material available online with this article; https://doi.org/10.1172/jci.insight.205826DS1). As expected from the DNCB irritant effect, the thickness of the ear and the epidermis and dermis, and the density of skin-infiltrating leukocytes, increased significantly in WT mice sensitized with DNCB, and these parameters were significantly lower in equally treated *Tac1^KO^* mice lacking SP (Figure 1, A–D, and Supplemental Figure 1A).

To determine whether the proinflammatory effects of SP were mediated via the NK1R (high-affinity SP receptor) or Mas-related G protein–coupled receptor B2 (Mrgb2) (low-affinity SP receptor), *NK1R^KO^*, *Mrgb2^KO^*, and WT mice were skin-sensitized with DNCB (right ear) or treated with vehicle (left ear), and the parameters of local innate immunity induced by the hapten were analyzed in the ear skin. The ear and epidermal-dermal thickness and the density of skin-infiltrating leukocytes of DNCB-treated ears were significantly reduced in *NK1R^KO^* mice compared with the values detected in *Mrgb2^KO^* mice or WT controls, and there were no significant differences between WT and *Mrgb2^KO^* mice (Figure 1, E–H).

Next, we analyzed whether the SP-NK1R axis was relevant not only to the induction of the innate immune response of ACD caused by DNCB, but also to that induced by skin exposure to chemicals that trigger irritant contact dermatitis (ICD). For these experiments, WT, *Tac1^KO^*, and *NK1R^KO^* mice were treated in the dorsal skin of the right ear with croton oil (CO; 2% in acetone). Evaluation of ear thickness 2, 4, 6, 8, and 24 hours after application of CO in WT mice showed significant increase, which peaked from 4 to 6 hours after treatment (Supplemental Figure 1B) and correlated with intense dermal edema and skin-infiltrating polymorphonuclear leukocytes. These parameters were significantly diminished in *Tac1^KO^* and *NK1R^KO^* mice evaluated up to 24 hours after treatment (Supplemental Figure 1, C–G).

Thus, the SP-NK1R axis is essential to initiate the local innate immunity required for the sensitization phase of ACD and the development of ICD.

### The SP-NK1R axis in keratinocytes is necessary for the proinflammatory skin environment and the adaptive immune response to DNCB.

Keratinocytes, the most abundant cells of the skin that are exposed to haptens, express the NK1R and are in contact with SP-secreting sensory nerves (18–22, 24, 50, 51). Therefore, we analyzed the role of SP-NK1R signaling specifically in keratinocytes during the initiation of the cutaneous innate immune response triggered by DNCB. To address that, *K14^Cre/+^ NK1R^KO^* mice, with specific deletion of the NK1R in keratinocytes (18), and their littermate (*Cre^neg^*) controls were treated topically with DNCB or vehicle on the dorsal skin of the right and left ear, respectively. Parameters indicative of development of an innate immune response were assessed in the ear skin 24 hours after sensitization. Compared with their littermate controls, *K14^Cre/+^ NK1R^KO^* mice exhibited significantly less increase of ear and epidermal-dermal thickness and of skin-infiltrating leukocytes in response to DNCB treatment (Figure 2, A–D). Consequently, compared with WT mice and their littermates, *K14^Cre/+^ NK1R^KO^* mice exhibited a significant decrease in the effector CHS response following skin sensitization and elicitation with DNCB, and this decrease was comparable to that observed in *Tac1^KO^* and global *NK1R^KO^* mice (Supplemental Figure 2, A–D).

The previous findings demonstrate that during the sensitization phase of ACD, SP exerts its proinflammatory function by signaling the NK1R. Therefore, we compared by Luminex the repertoire and concentration of proinflammatory mediators in the skin of *K14^Cre/+^ NK1R^KO^* mice and littermate controls 24 hours after topical application of DNCB or vehicle. Concentrations in skin tissue homogenates of IL-1β, IL-6, TNF-α, IL-15, IL-17, leukemia inhibitory factor (LIF), CCL2, CCL3, CCL4, CXCL1, CXCL4, CXCL10, GM-CSF, G-CSF, M-CSF, and VEGF were significantly decreased in the skin of DNCB-sensitized *K14^Cre/+^ NK1R^KO^* mice compared with the values observed in equally treated littermate controls (Figure 2E).

In summary, signaling of keratinocytes via the SP-NK1R axis is necessary and sufficient to generate the local proinflammatory environment that is essential for the sensitization and effector phases of ACD (10, 11, 14, 52–55).

### Signaling of keratinocytes via the SP-NK1R axis increases transcripts for inflammasome proteins.

Next, we investigated the mechanisms by which the SP-NK1R axis promotes the proinflammatory function of keratinocytes that initiates the sensitization of ACD. The role of TNF-α in the activation of the NLRP3 inflammasome resulting in secretion of IL-1β by keratinocytes has been shown to be essential for development of CHS to skin sensitizers, including DNCB (31–37). Thus, we analyzed whether SP increases expression of mRNA encoding TNF-α, and proteins that participate in priming (signal 1) and assembly (signal 2) of the NLRP3 inflammasome in keratinocytes. We used COCA cells, primary keratinocytes from the skin of adult mice (56) that express the functional variant of the NK1R (Supplemental Figure 3A). SP exerts its proinflammatory effects signaling via NK1R at concentrations ranging between 10^–6^ and 10^–9^ M (48, 57, 58). In our experimental conditions a dose-response experiment showed that 10^–7^ M was the optimal concentration of the synthetic stable form of SP, [Sar^9^,Met(O_2_)^11^]-substance P (SarSP), to induce pro–IL-1β and TNF-α transcripts in COCA cells (Supplemental Figure 3B). Likewise, treatment of COCA cells with SP or SarSP at a concentration of 10^–7^ M induced equivalent significant increases of pro–IL-1β, pro–IL-18, and pro–caspase-1 transcripts (Supplemental Figure 3C). Thus, the experiments in vitro were done with a SarSP concentration of 10^–7^ M.

Analysis by quantitative reverse transcription PCR (qRT-PCR) of the kinetics of expression of mRNA encoding components of the NLRP3 inflammasome following SarSP stimulation validated the induction of transcripts for pro–IL-1β, pro–IL-18, and pro–caspase-1, and also detected a significant increase of transcripts encoding other proteins involved in the NLRP3 inflammasome, including PyCard/ASC, NLRP3, P2x7R, and transient receptor potential vanilloid 1 (TRPV1) (Figure 3A) (59–61). P2x7R and TRPV1 are membrane receptors that participate in activation (signal 2) of the NLRP3 inflammasome (59, 61). In addition, we detected increased expression of mRNA for TNF-α, a cytokine that primes and sustains the activation of the NLRP3 inflammasome in aseptic conditions, and for IL-6, a proinflammatory cytokine that is increased by IL-1β and TNF-α (Figure 3A) (62–67).

Following skin penetration with haptens, IL-1β and TNF-α released locally initiate the inflammatory response required for the sensitization of ACD (10–14, 68). Thus, we verified that the increased expression of IL-1β and TNF-α mRNA detected by qRT-PCR in COCA cells treated with SarSP correlated with higher content of pro–IL-1β protein in cell lysates and of secreted TNF-α in culture supernatants of COCA cells treated with SarSP under the same conditions (Figure 3B).

Next, we analyzed whether the increased expression of mRNA for components of the NLRP3 inflammasome and for proinflammatory cytokines detected in keratinocytes treated with SarSP was mediated through signaling via NK1R. Analysis by qRT-PCR demonstrated that cultures of skin keratinocytes from *K14^Cre/+^ NK1R^KO^* mice, unlike those from littermate (*Cre^neg^*) controls, failed to upregulate the mRNA of pro–IL-1β, pro–caspase-1, TNF-α, and IL-6 in response to SarSP (Figure 3C). Thus, signaling of keratinocytes via the SP-NK1R axis augments expression of mRNA encoding proteins involved in priming and assembly of the NLRP3 inflammasome required for synthesis and secretion of IL-1β and increases synthesis and release of TNF-α — the 2 cytokines that initiate the cutaneous innate immune response during hapten sensitization (14, 69).

### The SP-NK1R axis induces pro–IL-1β and TNF-α in keratinocytes via different intracellular signaling pathways.

TNF-α primes and sustains the expression of the NLRP3 inflammasome under aseptic conditions and is an essential cytokine for the induction of ACD (63–67). Thus, we investigated whether pro–IL-1β in keratinocytes treated with SarSP is induced by the neuropeptide or indirectly through release of TNF-α. Comparative qRT-PCR analysis in cultures of keratinocytes obtained from WT or from *TNF-α^KO^* mice treated with SarSP or vehicle demonstrated that TNF-α is necessary to increase the expression of pro–caspase-1 and NLRP3 mRNA, but not pro–IL-1β mRNA (Figure 4A). Thus, SP-NK1R signaling of keratinocytes augments expression of pro–IL-1β in a TNF-α–independent fashion but increases pro–caspase-1 and NLRP3 transcripts in a TNF-α–dependent manner.

Next, we analyzed the intracellular pathways triggered by the SP-NK1R axis associated with the increase of expression of pro–IL-1β and TNF-α mRNA in keratinocytes. We investigated this in cultures of COCA cells treated with SarSP together with inhibitors of Gα_q/11_ (YM-254890), NF-κB (JSH-23), TNF-dependent NF-κB translocation (BAY11-7082), PI3K (CGS15943), calcium-dependent NFAT (VIVIT peptide), and the MAPK proteins JNKII (SP600125), p38 (SB203580), and ERK (PD98059). The qRT-PCR analysis showed that increase of TNF-α and pro–IL-1β transcripts by SarSP required signaling via Gα_q/11_, NF-κB, PI3K, and the MAPK proteins JNKII, p38, and ERK. Whereas increase of TNF-α transcripts depended on NFAT and TNF-dependent NF-κB translocation signaling, these pathways were not required for the increase of pro–IL-1β transcripts (Figure 4B).

Signaling via the NK1R induces intracellular Ca^2+^ increase in excitable and non-excitable cells, an effect that is needed for dephosphorylation and nuclear translocation of NFAT but is not necessarily required for NF-κB activation by SP (70–77). We confirmed in COCA cells that SarSP induced Ca^2+^ flux assessed by a ratiometric assay by FACS (Figure 4C), and nuclear translocation of NFAT and NF-κB, the latter inhibited by the mouse NK1R-specific antagonist RP67,580 (Figure 4D). However, SarSP stimulation of COCA cells in the presence of extracellular (EGTA) or intracellular (BAPTA-AM) Ca^2+^ chelators did not affect NF-κB nuclear translocation (Figure 4E).

In summary, these data confirm that SP-NK1R signaling in keratinocytes promotes per se the activation of key intracellular pathways, resulting in the synthesis of pro–IL-1β and TNF-α. In addition, the results show that SarSP increases the expression of pro–caspase-1 and NLRP3 transcripts in a TNF-α–dependent fashion.

### DNCB induces pro–IL-1β but not TNF-α in mouse and human keratinocytes.

The data to this point explain, at least in part, the molecular mechanisms by which the SP-NK1R axis promotes per se the proinflammatory function of keratinocytes, leading to the synthesis of pro–IL-1β and TNF-α, independently from each other. In addition, the data show that SarSP induces Ca^2+^ influx in mouse keratinocytes required for the NFATc1-dependent increase of TNF-α transcripts, and these effects were not necessary for the induction of pro–IL-1β transcripts. However, these findings do not elucidate the contribution of SP-NK1R signaling in the proinflammatory effect that DNCB exerts on keratinocytes during skin sensitization. Thus, we compared the proinflammatory effects that SarSP or DNCB exerts in COCA cells. DNCB (10 μM) increased NK1R expression as early as 2 hours after exposure of COCA cells (Figure 5A), but it did not induce intracellular Ca^2+^ increase (Figure 5B). Like SarSP, DNCB triggered nuclear translocation of NF-κB (Figure 5, C and D) and augmented expression of pro–IL-1β transcripts, and protein in cell lysates (Figure 5, G and I). Unlike SarSP, DNCB did not cause nuclear translocation of NFATc1 (Figure 5, E and F), increase of TNF-α mRNA, or the release of TNF-α protein in keratinocyte culture supernatants (Figure 5, H and J).

To assess the clinical translational relevance of our findings, we investigated in human keratinocytes to what extent the SP-NK1R axis plays a role in inducing the keratinocyte proinflammatory changes triggered by DNCB. For these experiments we stimulated HaCaT cells or primary keratinocytes from adult skin (HEKa cells) with SarSP or DNCB. Analysis of increase of transcripts for pro–IL-1β and TNF-α confirmed that stimulation of HaCaT cells with SarSP was optimal at a concentration of 10^–7^ M (Supplemental Figure 4A). Similarly to what we observed in mouse keratinocytes, stimulation of HaCaT cells with SarSP increased the transcripts for relevant proteins involved in the NLRP3 inflammasome, including pro–IL-1β, pro–IL-18, pro–caspase-1, NLRP3, and TRPV1, and for the proinflammatory cytokines TNF-α, IL-6, and IL-8 (Figure 6A).

HaCaT and HEKa cells expressed the functional variant of the NK1R, which was increased by treatment with DNCB (10 μM) for 2 hours (Figure 6B). Both DNCB and SarSP induced nuclear translocation of NF-κB and increase of pro–IL-1β transcripts (Figure 6, C and D). However, SarSP but not DNCB induced intracellular Ca^2+^ increase and nuclear translocation of NFATc1 (Figure 6, E and F). Consequently, only SarSP upregulated TNF-α mRNA in HaCaT and HEKa cells (Figure 6G). Importantly, nuclear translocation of NFAT and NF-κB was inhibited by the human NK1R–specific antagonist L733,060 (Supplemental Figure 4B).

### Administration of IL-1β plus TNF-α restores CHS in K14^Cre/+^ NK1R^KO^ mice.

The physiological relevance of the results obtained in vitro in mouse and human keratinocytes was assessed by comparison of the innate and adaptive immune responses to DNCB in *Cre^neg^* littermate controls and *K14^Cre/+^ NK1R^KO^* mice sensitized only or sensitized and elicited with topical DNCB, the latter in the presence or absence of intradermal (i.d.) IL-1β, TNF-α, or both. Controls included mice treated topically with the DNCB vehicle plus PBS or with DNCB vehicle and IL-1β plus TNF-α (i.d.).

Analysis of ear and epidermal-dermal thickness and the intensity of skin-infiltrating leukocytes confirmed that lack of NK1R in keratinocytes of *K14^Cre/+^ NK1R^KO^* mice prevented the innate immune response to topical DNCB in comparison with littermate (*Cre^neg^*) controls (Figure 7, A and B). The innate immune response to DNCB in *K14^Cre/+^ NK1R^KO^* mice was partially but significantly increased by i.d. injection of IL-1β or TNF-α in the sensitization site immediately after DNCB application, and it was fully restored and comparable to that of littermate controls following co-administration of both cytokines (Figure 7, A and B, and Supplemental Figure 5, A and B). As expected, i.d. administration of IL-1β and TNF-α in the absence of DNCB induced skin inflammation characterized by edema and inflammatory leukocyte infiltrate that peaked 24 hours after treatment, and these effects were significantly lower than the ones induced by skin co-administration of DNCB plus both cytokines (Figure 7A and Supplemental Figure 5, A and B).

Development of the adaptive immune response of ACD requires that skin-resident DCs migrate to the sdLNs to prime hapten-specific pathogenic T cells. Because (a) activation and traffic of skin-migratory DCs (smiDCs) depend on IL-1β and TNF-α released in the skin (10–12, 14, 15) and (b) expression of IL-1β and TNF-α mRNA in DNCB-sensitized skin is significantly lower in *K14^Cre/+^ NK1R^KO^* mice, we analyzed whether expression of NK1R in keratinocytes affects the number of smiDCs in sdLNs after DNCB sensitization. Flow cytometric analysis of sdLNs 72 hours after treatment showed that the number of total smiDCs identified by the phenotype CD45^pos^CD11c^pos^MHC-II^hi^ (Supplemental Figure 5C) was significantly decreased in *K14^Cre/+^ NK1R^KO^* mice compared with those in sdLNs of littermate controls (Figure 7C). Decrease of smiDCs in *K14^Cre/+^ NK1R^KO^* mice included epidermal Langerhans cells (LCs) (CD207^pos^CD103^neg^CD301b^neg^) and dermal type 1 cDCs (cDC1) (CD207^pos^CD103^pos^CD301b^neg^) and cDC2 (CD207^neg^CD103^neg^CD301b^pos^CD11b^hi^) (Figure 7D and Supplemental Figure 5D). In the absence of DNCB, i.d. IL-1β and TNF-α increased the number of smiDCs in the sdLNs of *K14^Cre/+^ NK1R^KO^* mice. However, only DNCB plus both cytokines restored the numbers of smiDCs in sdLNs to those observed in *Cre^neg^* littermate controls (Figure 7C and Supplemental Figure 5D). Likewise, the effector phase of CHS to DNCB was partially restored in *K14^Cre/+^ NK1R^KO^* mice injected i.d. with IL-1β or TNF-α at the DNCB sensitization and elicitation sites and was recovered to a level similar to that developed in littermate controls when both cytokines were co-administered with the hapten (Figure 7, E and F, and Supplemental Figure 5, E and F). As expected, i.d. administration of IL-1β and TNF-α in the absence of DNCB, analyzed 48 hours after elicitation, was not sufficient to stimulate the effector phase of the CHS (Figure 7, E and F, and Supplemental Figure 5, E and F).

In summary, lack of NK1R in keratinocytes is sufficient to prevent the skin innate and adaptive immune responses elicited by DNCB by a mechanism involving the synthesis and secretion of IL-1β and TNF-α.

## Discussion

The mechanisms behind the immunoregulatory function of neuropeptides released in the skin immediately after allergen exposure are currently the focus of intensive research for the development of immunotherapies to treat chronic and recurrent inflammatory skin disorders like ACD (6, 16, 18, 49, 78, 79). Several laboratories, including ours, have demonstrated the relevance of blocking the SP-NK1R axis to prevent the induction of CHS (6, 8, 18).

Keratinocytes are the main cell component of the epidermis and, together with cutaneous peripheral nerves, are first responders to harmful stimuli, including contact sensitizers (19–24). Interaction between keratinocytes and neuroinflammatory mediators like SP in the skin is critical for development of the innate immune response that leads to the effector phase of ACD (18). Nevertheless, gaps in the understanding of the mechanisms behind the neuroimmune regulation of keratinocyte function by contact sensitizers preclude development of treatments for ACD based on the neuroinflammatory pathogenesis of the disorder. Thus, the present study analyzed the cellular and molecular mechanisms by which the SP-NK1R axis is required for the proinflammatory function of keratinocytes exposed to haptens.

The inflammatory skin microenvironment required for efficient sensitization of ACD shares the mechanisms involved in the induction of ICD (80). Using global *Tac1^KO^* and *NK1R^KO^* mice, we demonstrate that lacking SP or the NK1R prevented both the innate immune response triggered by DNCB and that triggered by croton oil, indicating that the SP-NK1R axis operates at early time points following exposure to skin-contact sensitizers and irritants. As expected based on the impaired sensitization, the effector phase of the CHS to DNCB was significantly decreased in the absence of SP or NK1R.

Importantly, we demonstrated that the absence of NK1R exclusively in keratinocytes is sufficient to abrogate the innate and adaptive immune responses to DNCB to the same extent observed in global *Tac1^KO^* (lacking SP) and *NK1R^KO^* mice. These findings prove that the proinflammatory function of DNCB on the skin was mediated indirectly through the effect(s) of locally released SP signaling keratinocytes via the NK1R.

Mechanistic analysis through ex vivo quantification of cytokines, chemokines, and growth factors in the skin of *K14^Cre/+^ NK1R^KO^* mice sensitized with DNCB demonstrated significantly less content of (a) IL-1β and TNF-α, required for the activation and mobilization of smiDCs; (b) IL-6, IL-15, IL-17, LIF, and CXCL1, which promote skin homing of polymorphonuclear cells; (c) CCL2, CCL3, CCL4, and CXCL10, which induce skin infiltration by monocytes, macrophages, NK cells, and T cells; and (d) GM-CSF, G-CSF, M-CSF, and VEGF, which together support survival of myeloid cells, proliferation of vascular endothelial cells, and inflammation (52–55). Thus, following skin exposure to DNCB, signaling of keratinocytes via NK1R generates the inflammatory environment necessary for sensitization of CHS (53, 55).

Activation of the NLRP3 inflammasome resulting in secretion of IL-1β and IL-18 is necessary for induction of ACD by DNCB, TNCB, and metal contact sensitizers (31–37). Nevertheless, whether the SP-NK1R axis promotes the synthesis of proteins involved in the activation of the NLRP3 inflammasome in keratinocytes exposed to contact sensitizers remained to be completely elucidated. Using *K14^Cre/+^ NK1R^KO^* mice and littermate controls, we demonstrated that SP-NK1R signaling of keratinocytes induces a rapid increase in expression of transcripts encoding proteins involved in priming or assembly of the NLRP3 inflammasome, including pro–IL-1β, pro–IL-18, pro–caspase-1, NLRP3, PyCard/ASC, P2x7R, and TRPV1, as well as the proinflammatory cytokines IL-6 and TNF-α. Importantly, the upregulation of expression of pro–IL-1β and TNF-α mRNA correlated with increased concentrations of pro–IL-1β and TNF-α proteins at the skin sensitization site.

The NK1R is a G protein–coupled receptor that signals via Gα_q/11_ and G_s_ proteins (81). Signaling via Gα_q/11_ activates phospholipase C-β that induces inositol 1,4,5-triphosphate and diacylglycerol, resulting in intracellular Ca^2+^ mobilization (71, 81, 82). It also promotes nuclear translocation of NFATc1 in astrocytes, a transcription factor required for synthesis of TNF-α (71, 83, 84). Signaling via G_s_ proteins activates MAPK, protein kinase C, and cyclic adenosine monophosphate. Gα_q/11_, PI3K, and MAPK play a central role in the activation of the NLRP3 inflammasome, resulting in synthesis and release of proinflammatory mediators including pro–IL-1β and pro–IL-18 (63, 84). Here we showed that SP-NK1R signaling of keratinocytes increases expression of pro–IL-1β and TNF-α mRNA through the Gα_q/11_, PI3K, MAPK, and NF-κB pathways. These data agree with previous publications in a rat model of complex regional pain syndrome showing that the SP-NK1R axis induces synthesis of IL-1β and TNF-α in keratinocytes via JNK-MAPK (48, 49).

In our model, the increase of TNF-α mRNA in keratinocytes exposed to SarSP was mediated by activation of the Ca^2+^-dependent transcription factor NFATc1 and TNF-dependent activation of NF-κB, which were not required to induce the synthesis of pro–IL-1β mRNA. These results together with our finding that SarSP does not alter the expression of pro–IL-1β mRNA in *TNF-α^KO^* keratinocytes indicate that SP-NK1R signaling of keratinocytes is per se sufficient to increase pro–IL-1β and TNF-α, independently of each other.

IL-1β and TNF-α work in concert to initiate and amplify the skin inflammation that leads to the adaptive immune response of ACD (9–11, 13, 14, 85). Our results showed that DNCB and SarSP induced, in mouse keratinocytes, NF-κB nuclear translocation and increased mRNA expression and synthesis of pro–IL-1β. However, unlike SarSP, DNCB did not cause intracellular Ca^2+^ increase, translocation of NFATc1, or induction of TNF-α mRNA and synthesis.

Similarly to the data obtained in mouse models, SP-NK1R signaling of human HaCaT and HEKa cells showed that SarSP increased the transcripts for proteins involved in the NLRP3 inflammasome, including pro–IL1β, TNF-α, IL-6, and the human proinflammatory cytokine IL-8. DNCB upregulated the functional NK1R variant in both cell types and increased NF-κB and pro–IL1β transcripts, but only SarSP triggered intracellular Ca^2+^ increase and NFATc1 translocation and induced TNF-α mRNA. The fact that the human-specific NK1R antagonist L733,060 blocked NFAT and NF-κB translocation confirmed a mechanism mediated via the NK1R. Together the data confirm that the molecular mechanisms of SP-NK1R signaling in keratinocytes are conserved between mouse and humans, validating the translational relevance of our results.

Effective sensitization of ACD depends on mobilization and homing in sdLNs of smiDCs transporting haptenated proteins, a function that requires secretion of IL-1β and TNF-α in the skin (9–11, 13, 14, 86). Our results in vivo demonstrated that efficient mobilization of skin-resident LCs, cDC1, and cDC2 in mice sensitized with DNCB requires expression of NK1R in keratinocytes. As expected, i.d. administration of IL-1β plus TNF-α at the site of DNCB sensitization in *K14^Cre/+^ NK1R^KO^* mice restored the mobilization of smiDCs to sdLNs and the effector phase of CHS. Interestingly, although DNCB increased pro–IL-1β levels in keratinocytes, co-administration of topical DNCB with i.d. TNF-α did not restore the CHS response. This could be due to the kinetics, dosage, or interaction of these cytokines, or to defective synthesis of NLRP3 and/or pro–caspase-1, which, under our experimental conditions, required TNF-α. Analysis of these mechanisms, however, exceeds the scope of the present work.

Recent studies showed that the irritant effect of DNCB induces secretion of SP by skin sensory nerves via activation of the transient receptor potential ankyrin 1 (TRPA1) channel (38, 40–43). In combination with the results reported here, we propose a model in which, following DNCB sensitization, SP released locally induces via NK1R a proinflammatory phenotype in keratinocytes. This was sufficient by itself and essential for triggering cutaneous inflammation and the subsequent adaptive immune response of CHS.

In summary, our results unveil, at least in part, the molecular and cellular mechanisms by which SP-NK1R signaling in keratinocytes initiates the proinflammatory skin environment triggered by contact sensitizers. We show that the axis functions early following hapten penetration in the skin to signal in keratinocytes the intracellular pathways required for the synthesis of TNF-α, and activates the NLRP3 inflammasome, resulting in secretion of IL-1β, which provides the skin inflammatory environment required for the mobilization of smiDCs, known to transport hapten-modified proteins/peptides to the sdLNs and initiate the adaptive immune response, accounting for the effector phase of ACD (12, 18, 69, 87).

Because the molecular mechanisms involved in signaling of keratinocytes via the SP-NK1R axis function at the initiation of the sensitization phase, it represents a cell type–specific target for future immunotherapies. Further studies on SP-NK1R signaling in keratinocytes in patients with ACD will translate these findings into treatments that target skin neuroinflammatory networks.

## Methods

### Sex as a biological variable.

Our study examined male and female animals, and similar findings are reported for both sexes.

### Mice.

Male and female mice (7–12 weeks old) were randomly selected for the experiments in accordance with the NIH scientific rigor policy. Mice were maintained in the pathogen-free animal facility of the University of Pittsburgh School of Medicine, which provides around-the-clock husbandry and veterinary services. C57BL/6 (B6), B6.Cg-*Tac1^tm1Bbm^*/J (*Tac1^KO^*), B6N.Cg-Tg(*KRT14-cre*)1Amc/J (K14-Cre), and B6.129S-*Tnf^tm1Gkl^*/J (*TNF-α^KO^*) mice were purchased from The Jackson Laboratory. Homozygous B6 *NK1R^KO^* mice were provided by C.J. Paige (University of Toronto, Toronto, Ontario, Canada) (88–90), B6 *Mrgb2^KO^* mice were provided by B.D. McNeil (Northwestern University, Chicago, Illinois, USA) (91), and B6 *NK1R^fl/fl^* mice were provided by N.L. Ward (Vanderbilt University Medical Center, Nashville, Tennessee, USA). B6 *K14^Cre/+^ NK1R^KO^* mice were generated in our laboratory as previously published (18).

### Cultures of mouse and human keratinocytes.

COCA cells (keratinocytes from C57BL/DBA adult mice generated in vitro by several culture passages) were purchased from the European Collection of Authenticated Cell Cultures and cultured in CnT-07 medium (CELLnTEC) containing 0.2 mM CaCl_2_ during the first 24 hours and 0.07 mM CaCl_2_ for the following days (56).

Primary cultures of mouse keratinocytes were generated from the tail skin digested in Keratinocyte Basal Medium (EpiLife, Thermo Fisher Scientific) containing Dispase II (4 mg/mL; Sigma-Aldrich), and incubated overnight at 4°C. Next, the epidermis was separated from the dermis with fine forceps and further digested with TrypLE medium (Thermo Fisher Scientific) containing trypsin (0.25%; Sigma-Aldrich) for 20 minutes at room temperature, under continuous agitation, to obtain a single-cell suspension. Keratinocytes were cultured in Keratinocyte Basal Medium with EpiLife Defined Growth Supplement (Thermo Fisher Scientific).

HaCaT cells (human spontaneously transformed keratinocytes) were purchased from Applied Biomedical Materials and cultured in DMEM (HyClone, Cytiva SH30585.02) supplemented with CaCl_2_ (0.03 mM), 10% chelexed FBS (Gibco), and l-glutamine (HyClone SH30034.01). HEKa cells (primary human epidermal keratinocytes isolated from adult skin) were purchased from Gibco and cultured in EpiLife Medium supplemented with HKGS. All cells were tested for mycoplasma before use.

### Treatment of keratinocyte cell cultures.

Mouse and human keratinocytes were treated once the cultures reached about 80% of cell confluence. Keratinocytes were treated with SP (Tocris) or with the SP synthetic analog [Sar^9^,Met(O_2_)^11^]-substance P (SarSP; Tocris), both at 10^–7^ M unless otherwise indicated, or with DNCB (10 μM; Sigma-Aldrich), added at indicated time points. In vitro blockade of the NK1R was achieved using the mouse NK1R–specific inhibitor RP67,580 (10^–5^ M) or the human NK1R–specific inhibitor L733,060 (10^–5^ M), both from Tocris.

For analysis of intracellular signaling pathways, the following inhibitors or corresponding vehicles were added to the keratinocyte cell cultures 20 minutes before SarSP treatment: (a) YM-254890 (Gα_q/11_ inhibitor, 10 μM); (b) BAY11-7082 (inhibitor of TNF-dependent NF-κB-translocation, 5 μM); (c) CGS15943 (PI3K inhibitor, 10 μM); (d) SP600125 (JNKII inhibitor, 20 μM); (e) SB203580 (p38 inhibitor, 10 μM); (f) JSH-23 (NF-κB inhibitor, 20 μM) (all from Tocris); (g) VIVIT peptide (NFAT Inhibitor-1; 5 μM); and (h) PD98059 (MEK-ERK inhibitor, 30 μM) (both from MedChemExpress). Ca^2+^-dependent intracellular signaling pathways were inhibited with EGTA (1 mM) or with BAPTA-AM (10 μM) (both from Sigma-Aldrich).

### Induction of cutaneous CHS and ICD.

To assess the cutaneous innate immune response during the sensitization phase of CHS, mice were treated with 50 μL of 2,4-dinitrochlorobenzene (DNCB) (1% in 1:4 vol/vol acetone/olive oil [A/OO]) applied topically on the dorsal skin of the right ear or with A/OO applied on the dorsal skin of the left ear (controls).

For analysis of the effects of IL-1β and TNF-α in the innate immune response of CHS in the absence of NK1R in keratinocytes, *K14^Cre/+^ NK1R^KO^* and their littermate (*Cre^neg^*) control mice were sensitized as described above, and *K14^Cre/+^ NK1R^KO^* mice were injected i.d. in the sensitization site with IL-1β (25 ng), TNF-α (50 ng), or both in 50 μL of PBS, or with PBS alone (controls).

To assess the cutaneous adaptive immune response of CHS, mice were treated with 50 μL of DNCB (1% in A/OO) applied topically on the shaved skin of the lower abdomen. Five days after, mice were challenged with 50 μL of DNCB (0.5% in A/OO) applied topically on the dorsal skin of the right ear. As negative controls, mice were treated on the abdominal skin with A/OO and challenged on the dorsal skin of the left ear with A/OO.

For analysis of the effects of IL-1β and TNF-α on the adaptive immune response of CHS in the absence of NK1R in keratinocytes, *K14^Cre/+^ NK1R^KO^* and their corresponding *Cre^neg^* littermate control mice were sensitized and elicited with DNCB as described above. *K14^Cre/+^ NK1R^KO^* mice were injected i.d. in the sensitization and elicitation sites with IL-1β, TNF-α, or both. Controls included *K14^Cre/+^ NK1R^KO^* and *Cre^neg^* mice treated topically on the skin with DNCB vehicle (A/OO) and injected i.d. with PBS, or treated topically with vehicle (A/OO) and injected i.d. with IL-1β plus TNF-α.

Ear thickness was evaluated 24, 48, and 72 hours after sensitization and elicitation using a digital micrometer (Mitutoyo). Values are expressed as percentage of ear thickness increase obtained by the formula [(thickness of treated ear – thickness of untreated ear) / (thickness of untreated ear) × 100]. Representative skin sections were obtained at the peak of the innate immune response (24 hours after skin sensitization) or at the peak of the effector CHS response (48 hours after elicitation) and processed for histological evaluation.

To assess the role of the SP-NK1R axis in the induction of ICD, WT, *Tac1^KO^*, and *NK1R^KO^* mice were treated on the dorsal skin of the right ear with croton oil (CO; Sigma-Aldrich) 2% in acetone. As control, the left ear was treated with CO vehicle. Evaluation of ear thickness was performed 2, 4, 6, 8, and 24 hours after application of CO using a digital micrometer (Mitutoyo). Representative skin sections were obtained at the peak of the inflammatory response (6 hours after CO application) and processed for histological evaluation.

### Quantification of skin-infiltrating leukocytes and measurement of epidermis and dermis thickness.

Mouse ear samples were dissected at the indicated time points, fixed in 4% paraformaldehyde, and embedded in paraffin, and tissue sections were stained with hematoxylin and eosin (H&E). Images of vertical sections of the ears were acquired with a Zeiss Axiovert-135 microscope equipped with a charge-coupled device (CCD) camera (Photometrics CH 250, Teledyne Imaging). Quantification of leukocytes was done in 20 high-power fields (HPFs; ×500) per skin section using AxioVision software (Carl Zeiss Vision). Epidermal-dermal thickness was measured on digital images of skin vertical sections using the measure tool of the AxioVision software.

### Analysis by qRT-PCR of mouse and human keratinocytes.

For quantification of mRNA encoding inflammasome subunits and cytokines, mouse keratinocytes including primary cultures of keratinocytes and COCA cells, and human keratinocytes including HaCaT and HEKa cells, were treated with SarSP (10^–7^ M), DNCB, or their vehicles. At the indicated time points, mRNA was extracted using TRIzol Reagent (Thermo Fisher Scientific) and quantified using a spectrophotometer (DeNovix DS-11). Two micrograms of RNA was reverse-transcribed to cDNA using the QuantiTect Reverse Transcription Kit (QIAGEN), and qRT-PCR was performed using TaqMan Fast Advanced Mastermix (Applied Biosystems) according to the manufacturer’s protocols. For mouse keratinocytes the following TaqMan real-time PCR assays (Applied Biosystems) were used: pro–IL-1β (Mm.PT.58.00434228 m1, FAM-MGB dye), TNF-α (Mm.PT.58.12575861), pro–IL-18 (Mm.PT.58.9981538), pro–caspase-1 (Mm.PT.58.13005595), PyCard/ASC (Mm.PT.56a.42872867), NLRP3 (Mm.PT.58.13974318), P2x7R (Mm.PT.58.31526331), TRPV1 (Mm.PT.58.13426135), IL-6 (Mm.PT.58.00446190 m1, FAM-MGB dye), and β-actin (Mm.PT.58.00443112, VIC-MGB PL).

For human keratinocytes, the following TaqMan real-time PCR assays were used: pro–IL-1β (Hs01555410_m1, FAM-MGB), pro–IL-18 (Hs.PT.58.25675872), pro–caspase-1 (Hs.PT.58.00354836), NLRP3 (Hs.PT.58.393003321), TRPV1 (Hs.PT.58.00218912_m1), IL-6 (Hs.PT.58.00174131_m1), IL-8 (Hs.PT.58.39926886), and HPRT1 (Hs.PT.58v.456221572). Multiplex reactions for human and mouse keratinocyte samples were run and analyzed using a StepOnePlus Real-Time PCR System (Applied Biosystems). Relative fold changes of individual cytokines were normalized to endogenous control and calculated based on the 2^−ΔΔCt^ method.

### Analysis of nuclear translocation of transcription factors by fluorescence confocal laser scanning microscopy and ELISA-based assay.

A complete list of antibodies used in the present study, specifying name, clone, provider, and catalog number, is provided in Supplemental Table 1.

To analyze nuclear translocation of NFATc1 and NF-κB by fluorescence confocal laser scanning microscopy (confocal microscopy), HaCaT cells were cultured in serum starvation conditions (serum-free DMEM) for 48 hours, a procedure that removes the background interference caused by growth factors and cytokines present in the FBS. Next, 1.5 × 10^3^ COCA or HaCaT cells were cultured over sterile round coverslips (18 mm; no. 1.5, Epredia) placed in 12-well plates (Corning, Fisher Scientific) for 24 hours and then treated with SarSP (10^–7^ M, 30 minutes) or DNCB (10 μM, 2 hours) or with DMSO (DNCB vehicle, 1 μL/mL). Cells were then fixed in 4% paraformaldehyde for 10 minutes, permeabilized with 0.1% Triton X-100 for 10 minutes, and blocked with 1% BSA for 45 minutes. Cells were then labeled with either rabbit polyclonal NF-κB p65 antibody (Ab) or mouse-monoclonal NFATc1 Ab, both at a concentration of 1:300 (overnight, 4°C), followed by secondary anti-rabbit or anti-mouse Alexa Fluor 488 Abs. F-actin was stained with Alexa Fluor 647–phalloidin at a concentration of 1:300, and nuclear stain was done with 4′,6-diamidino-2-phenylindole (DAPI; 300 nM in PBS). Images were acquired using a Nikon A1 confocal microscope equipped with a CCD camera. Nuclear translocation of NF-κB and NFATc1 was quantified using ImageJ/Fiji software (NIH). A total of 20 cells across 8 HPFs were selected based on a clearly defined DAPI signal and absence of mitosis or cell overlapping. Nuclear regions of interest (ROIs) were delineated based on DAPI staining, and the corresponding cytoplasmic ROI was drawn adjacent to the nucleus. The mean fluorescence intensity (MFI) was measured within each ROI, and nuclear translocation was expressed as the nuclear-to-cytoplasmic MFI ratio per cell.

To analyze nuclear translocation of NF-κB p65 and NFATc1 in nuclear extracts of COCA and HaCaT cells, we used TransAM enzyme-linked immunosorbent assay–based (ELISA-based) kits specific for human and mouse activated transcription factors (Active Motif). Cells were treated with SarSP (10^–7^ M) or DNCB (10 μM) or DNCB vehicle (DMSO 1 μL/mL) for 1 hour or 3 hours for analysis of nuclear translocation of NFATc1 and NF-κB p65, respectively. Nuclear extracts were obtained according to the manufacturer’s protocol, and the total nuclear proteins were incubated (40 μg/well) in 96-well plates coated with immobilized oligonucleotides containing the activated consensus sequences: 5′-AGGAAA-3′ for NFATc1 or 5′-GGGACTTTCC-3′ for NF-κB p65. Quantification of activated transcription factors was performed according to manufacturer’s protocol.

### Detection of cytokine proteins by ELISA.

For quantification of mouse pro–IL-1β and TNF-α protein by ELISA, 2 × 10^6^ COCA cells per experimental variable were treated with SarSP (10^–7^ M), DNCB (10 μM), or their vehicles. At indicated time points, pro–IL-1β was quantified in cell lysates using mouse pro–IL-1β precoated plates and kit (Biomatik). Mouse TNF-α was quantified in cell culture supernatants using mouse TNF-α high-sensitivity precoated plates and kit (Invitrogen), according to the manufacturer’s protocols.

### Ca^2+^ flux assays.

Ratiometric analysis of Ca^2+^ flux was done by FACS in COCA and HaCaT cells resuspended in PBS without Ca^2+^ and Mg^2+^ and loaded with Fluo-4 AM (2 μM) and Fura Red-AM Cell Permeant (2 μM) (both Invitrogen) for 30 minutes, in the dark, at 37°C. After thorough washing of the cells with PBS without Ca^2+^ and Mg^2+^, 2 × 10^6^ cells were resuspended in 1 mL of PBS containing 25 mM HEPES, 0.9 mM Ca^2+^, 0.5 mM Mg^2+^, and 1 mM sodium pyruvate. Five minutes before recording by FACS, the tubes containing COCA or HaCaT cells were warmed up in a water bath at 37°C, a temperature that was maintained throughout the recording time. Cells were analyzed immediately with BD LSR II or Fortessa flow cytometers. After recording of the 30-second baseline, cells were treated with SarSP (10^–7^ M) or with DNCB (10 μM), and data were recorded for 5 minutes. The ratiometric Fluo-4 AM/Fura Red-AM analysis was performed using the derived parameter of FlowJo v10 software (Tree Star, Becton Dickinson), and the kinetics tool was used to represent the Ca^2+^ flux graphically. Statistical comparison of Ca^2+^ flux from independent samples was achieved by comparison of the area under the curve per variable with the kinetics analysis function of the FlowJo v10 software.

### Quantification of cytokines in skin by Luminex.

Mice treated 24 hours earlier with DNCB or vehicle on the shaved skin of the abdomen were euthanized, and biopsies (~3 mm diameter) of the treated skin were excised using a disposable skin biopsy punch. Skin fragments were minced with a scalpel blade and transferred to Navy RINO tubes (Next Advance) in 425 μL of T-PER Tissue Protein Extraction Reagent (Thermo Fisher Scientific) containing 1× Protease Inhibitor Cocktail Kit (Thermo Fisher Scientific). Next, samples were homogenized with a Bullet Blender 5 Storm (Next Advance) at 4°C and standardized according to the protein concentration. Luminex Corporation xMAP technology was used to measure 32 mouse cytokines, chemokines, and growth factors using the Eve Technologies Mouse Cytokine/Chemokine 32-Plex Discovery Assay Array (MD32), according to the manufacturer’s instructions. Assay sensitivities ranged from 0.3 to 30.6 pg/mL.

### FACS analysis of NK1R in mouse and human keratinocytes.

Keratinocytes incubated with DNCB (10 μM) for 2 hours or left untreated were rinsed in PBS containing 1% FBS. To detect the expression of the NK1R on the cell surface, cells were incubated with an APC-conjugated NK1R Ab recognizing the N-terminus of the human and mouse NK1R (rabbit polyclonal, Novus Biological). To detect the intracellular expression of the NK1R, keratinocytes were rinsed in PBS, fixed in 2% paraformaldehyde (15 minutes, room temperature), and permeabilized with 0.1% saponin and 1% FBS in PBS (10 minutes, room temperature). Next, cells were incubated with a PE-conjugated Ab against the intracellular domain (C-terminus) of human and mouse NK1R (1:200, 30 minutes, 4°C). Then, cells were rinsed in permeabilization buffer, resuspended in PBS containing 1% FBS, and immediately analyzed with a BD LSR II flow cytometer. Dead cells were excluded by labeling with eFluor 780 Fixable Viability Dye (e-780-FVD). Results were analyzed with FlowJo v10 software. Fluorochrome-conjugated species- and isotype Ig–irrelevant Abs were used as staining controls.

### Quantification of mouse smiDCs in sdLNs.

*K14^Cre/+^ NK1R^KO^* mice or their littermate controls (*Cre^neg^*) were sensitized on the lower abdominal skin with 100 μL of DNCB in A/OO and then left untreated or injected i.d. with recombinant mouse IL-1β (25 ng), TNF-α (50 ng), or both in 50 μL of PBS, immediately after DNCB application. Seventy-two hours later, mice were euthanized, and single-cell suspensions of sdLN (inguinal) were stained with PE-CD45.2, PE-Cy7-IA^b^, BV510-CD11c, BV421-CD11b, APC-CD207, BUV395-CD103, and PerCP-Cy5.5-CD301b Abs, followed by FACS analysis. In all the experiments, fluorochrome-conjugated species- and isotype Ig–irrelevant Abs were used as staining controls, and dead cells were excluded by labeling with e-780-FVD. Absolute cell numbers were calculated by addition of CountBright Absolute Counting Beads to the samples immediately before FACS analysis.

### Statistics.

Statistical analyses were done using GraphPad Prism v9 software. Results are expressed as mean ± 1 SD. Comparisons of 2 means were done by 2-tailed Student’s *t* test. Comparison of multiple means on a single data set was done by 1-way ANOVA and post hoc Student-Newman-Keuls test. A *P* value less than 0.05 was considered significant.

### Study approval.

Animal care and handling were carried out in accordance with institutional guidelines and the procedures approved by University of Pittsburgh IACUC protocol 24105723.

### Data availability.

All data supporting the findings of this study are available within the article and its supplemental materials. Values underlying all graphs and summary statistics in the article are provided in the Supporting Data Values file. This study did not generate large datasets requiring deposition in a public repository. Any additional data are available upon reasonable request.

## Author contributions

SM and MB conducted in vitro and in vivo experiments, analyzed data, and contributed with manuscript writing. OAT performed cell purification and cultures, FACS analysis, and in vivo experiments. WJS carried out tissue dissection and sectioning, staining of tissue sections, and tissue imaging. GAG and SCW performed confocal microscopy and image acquisition and helped with interpretation of the results. AEM planned and conducted in vitro and in vivo experiments, analyzed data, and contributed with manuscript writing and intellectual input. ATL planned and supervised the experiments, conducted FACS analysis and in vivo experiments, and coordinated the writing of the manuscript.

## Conflict of interest

The authors have declared that no conflict of interest exists.

## Funding support

This work was supported in whole by NIH funding and is subject to the NIH Public Access Policy. Through acceptance of this federal funding, the NIH has been given a right to make the work publicly available in PubMed Central.

- NIH grants R01AI168142 to ATL and R01AI174273 to AEM.

## Supplementary Material

Supplemental data

Supporting data values

## Figures and Tables

**Figure 1:**
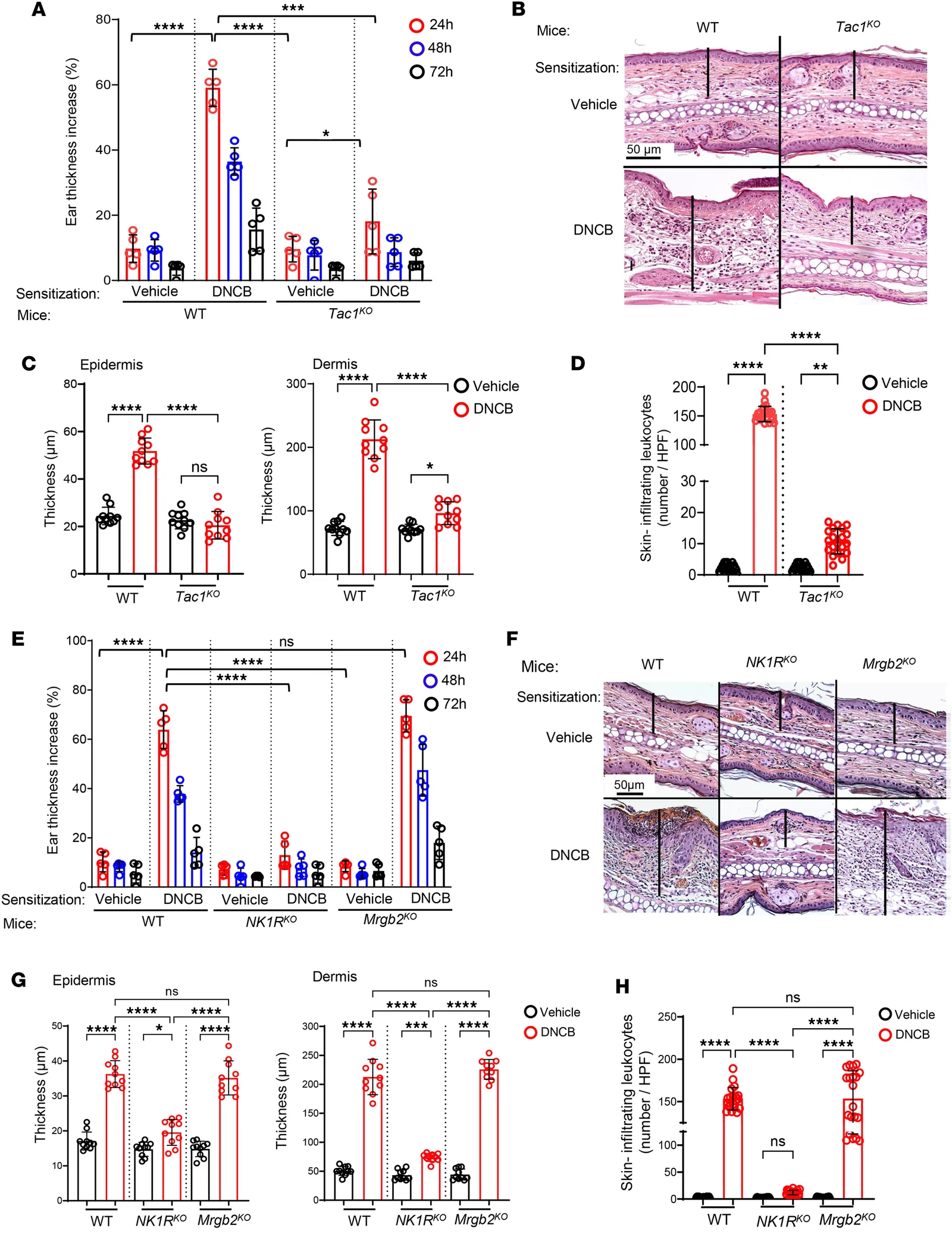
SP and the NK1R are necessary for the skin innate immunity triggered by topical exposure to DNCB. (**A**) Skin innate immune response to topical DNCB by percentage of ear thickness increase in WT and *Tac1^KO^* mice. (**B**) Microscopic images of ear sections of mice in **A** showing epidermal-dermal thickness (vertical lines) and infiltrating leukocytes. Scale bar: 50 μm. (**C** and **D**) Quantification of epidermal and dermal thickness (**C**) and infiltrating leukocytes (**D**) in skin sections shown in **B**. (**E**) Innate immune response to DNCB by percentage of ear thickness increase in WT, *NK1R^KO^*, and *Mrgb2^KO^* mice. (**F**) Microscopic images of ear sections of mice in **E** showing epidermal-dermal thickness (vertical lines) and infiltrating leukocytes. Scale bar: 50 μm. (**G** and **H**) Quantification of epidermal and dermal thickness (**G**) and infiltrating leukocytes (**H**) in the skin sections shown in **F**. In **A** and **E**, each circle represents 1 mouse (*n* = 5). **B** and **F** are images of samples obtained 24 hours after sensitization. H&E; original magnification, ×200. In **C** and **G**, each circle represents 1 measurement (*n* = 10). In **D** and **H**, each circle represents the quantification of skin leukocytes in 1 microscopic high-power field (HPF), ×500 (*n* = 20). Means ± 1 SD were analyzed by 1-way ANOVA and post hoc Student-Newman-Keuls test; *P < 0.05, **P < 0.01, ***P < 0.001, ****P < 0.0001.

**Figure 2:**
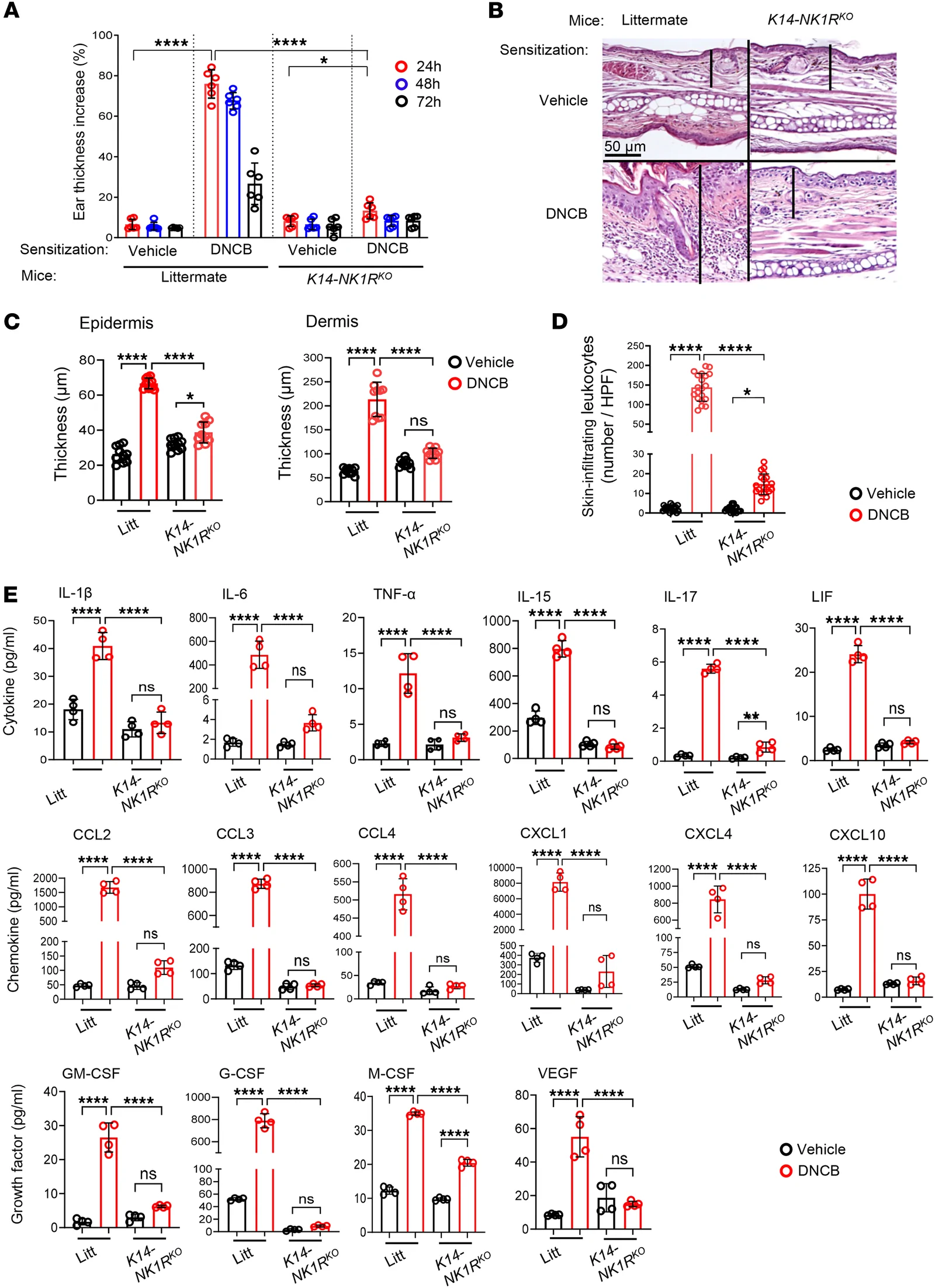
Expression of NK1R in keratinocytes is required for the skin inflammatory environment triggered by topical DNCB. (**A**) Skin innate immune response to DNCB in *K14^Cre/+^ NK1R^KO^* (*K14-NK1R^KO^*) mice and their littermate (Litt) controls assessed by percentage of ear thickness increase. (**B**) Microscopic images of the ears of mice in **A** showing epidermal-dermal thickness (vertical lines) and skin-infiltrating leukocytes. Scale bar: 50 μm. (**C** and **D**) Quantification of epidermal-dermal thickness (**C**) and infiltrating leukocytes (**D**) in the skin sections of *K14-NK1R^KO^* and littermate (Litt) controls shown in **B**. (**E**) Quantification by Luminex of proinflammatory mediators in skin samples of mice treated as in **A**. In **B**, images are from samples obtained 24 hours after sensitization. H&E; original magnification, ×200. In **A**, each circle represents 1 mouse (*n* = 6). In **C**, each circle represents 1 measurement (*n* = 10). In **D**, each circle represents quantification of skin-infiltrating leukocytes in 1 microscopic HPF, ×500 (*n* = 10). In **E**, each circle represents 1 reading of a skin sample from 1 mouse (*n* = 4). Means ± 1 SD were analyzed by 1-way ANOVA and post hoc Student-Newman-Keuls test; *P < 0.05, **P < 0.01, ****P < 0.0001.

**Figure 3:**
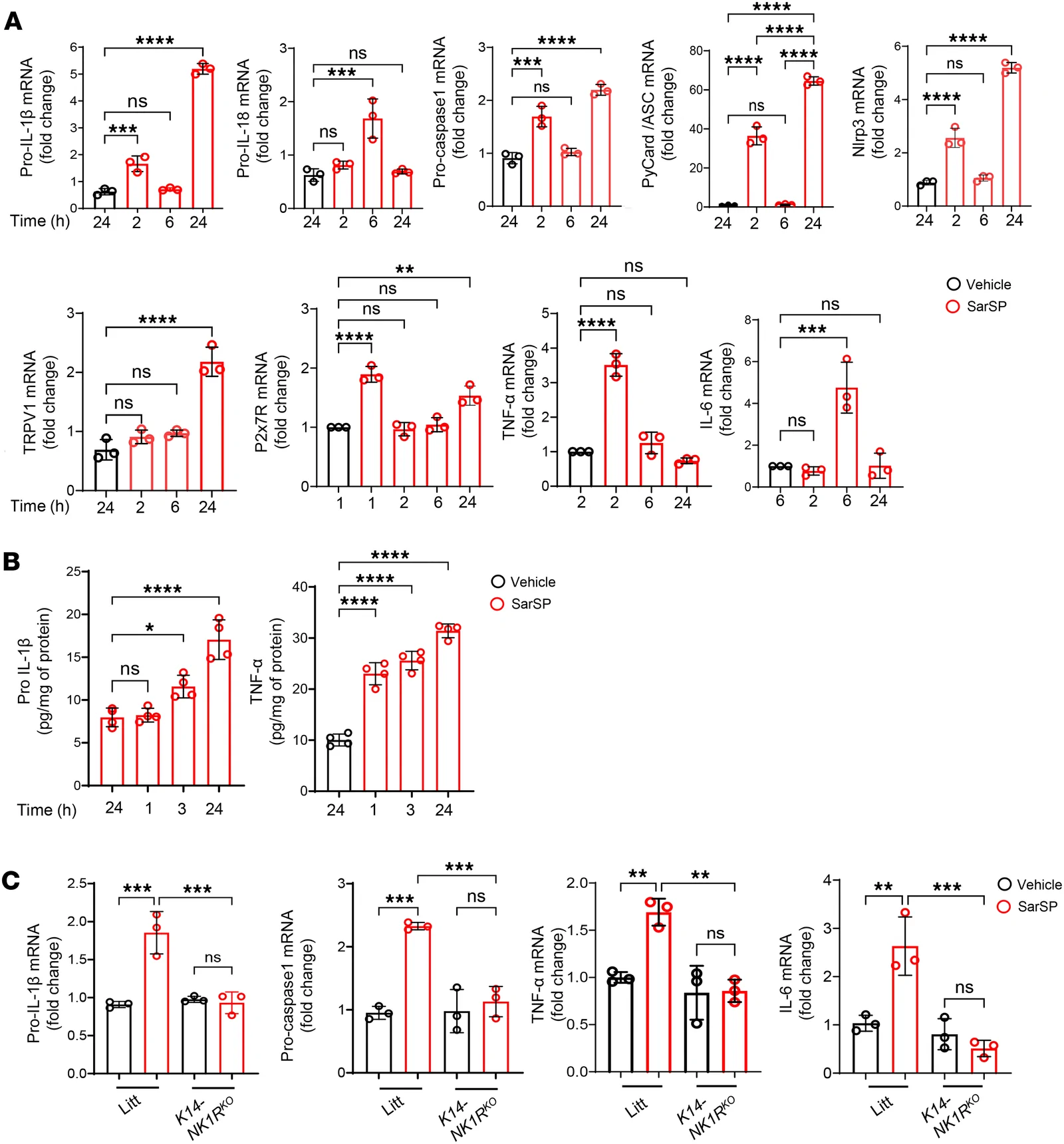
SP-NK1R signaling increases transcripts for proteins of the NLRP3 inflammasome and synthesis of pro–IL-1β and TNF-α in mouse keratinocytes. (**A**) Quantification by qRT-PCR of transcripts for proteins of the NLRP3 inflammasome in COCA cells at indicated times following treatment with SarSP (10^–7^ M) or vehicle. (**B**) Detection of pro–IL-1β and TNF-α protein by ELISA in COCA cells at indicated times following treatment with SarSP (10^–7^ M) or vehicle. (**C**) Comparative analysis by qRT-PCR of pro–IL-1β, pro–caspase-1, TNF-α, and IL-6 transcripts in primary cultures of keratinocytes isolated from *K14^Cre/+^ NK1R^KO^* (*K14-NK1R^KO^*) or littermate (Litt) control mice. In **A** and **B**, each circle represents 1 culture of COCA cells (*n* = 3 and *n* = 4, respectively). In **C**, each circle represents a primary keratinocyte culture from 1 mouse (*n* = 3). Means ± 1 SD were analyzed by 1-way ANOVA and post hoc Student-Newman-Keuls test; *P < 0.05, **P < 0.01, ***P < 0.001, ****P < 0.0001.

**Figure 4:**
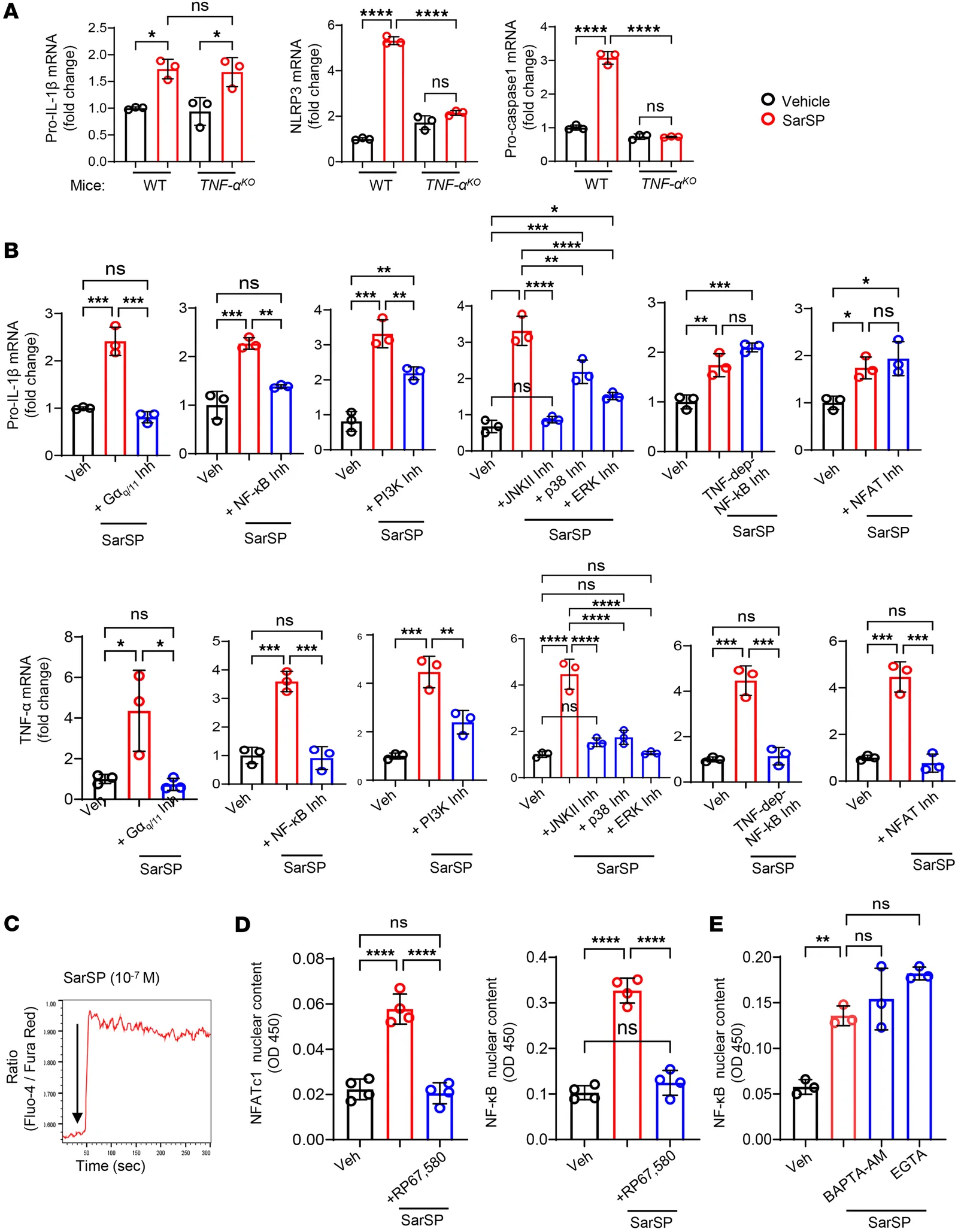
SarSP stimulates intracellular signaling pathways involved in synthesis of pro–IL-1β and TNF-α. (**A**) Quantification by qRT-PCR of transcripts for pro–IL-1β, NLRP3, and pro–caspase-1 in primary cultures of keratinocytes isolated from WT or *TNF-α^KO^* mice. (**B**) Analysis by qRT-PCR of the intracellular signaling pathways involved in the increase of pro–IL-1β and TNF-α transcripts in COCA cells treated with SarSP (10^–7^ M) alone or with intracellular pathway inhibitors or their vehicles (Veh). (**C**) FACS analysis of Ca^2+^ flux induced by SarSP (10^–7^ M) in COCA cells. A representative experiment of 3 is shown. (**D**) Quantification by ELISA-based assay of NFATc1 and NF-κB nuclear translocation in COCA cells treated with SarSP (10^–7^ M) alone or with the mouse NK1R-specific antagonist RP67,580 (10^–5^ M) or vehicle. (**E**) Quantification by ELISA-based assay of NF-κB nuclear translocation in COCA cells treated with SarSP (10^–7^ M) alone or with intracellular (BAPTA-AM) or extracellular (EGTA) Ca^2+^ chelators. In **A**, each circle represents a keratinocyte primary culture from 1 mouse (*n* = 3). In **B**, **D**, and **E**, each circle represents a single culture of COCA cells (**B**, *n* = 3; **D**, *n* = 4; **E**, *n* = 3). Means ± 1 SD were analyzed by 1-way ANOVA and post hoc Student-Newman-Keuls test; *P < 0.05, **P < 0.01, ***P < 0.001, ****P < 0.0001.

**Figure 5:**
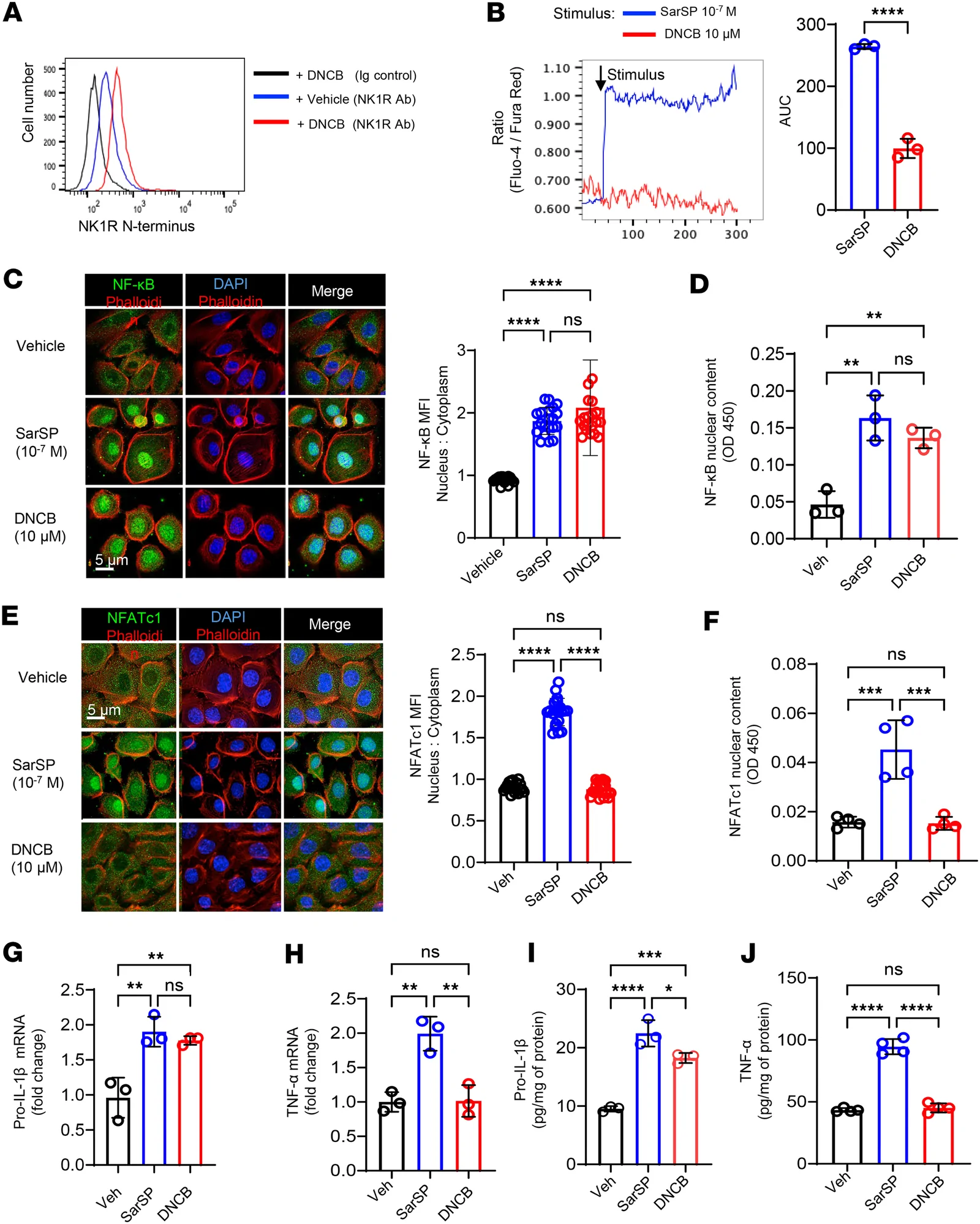
Synthesis of pro–IL-1β and TNF-α in mouse keratinocytes stimulated with DNCB or SarSP. (**A**) Expression (FACS) of the N-terminus of NK1R in COCA cells stimulated with DNCB (10 μM, 2 hours) or vehicle. (**B**) Ca^2+^ flux (FACS) induced by SarSP or DNCB in COCA cells. Left: one experiment of 3. Right: Comparison of Ca^2+^ flux by calculation of the area under the curve (AUC) (with FlowJo). Each circle represents 1 cell culture (*n* = 3). (**C** and **E**) Left: Representative images obtained by confocal microscopy of NF-κB (**C**) or NFATc1 (**E**) nuclear translocation in COCA cells treated with SarSP, DNCB, or DNCB vehicle (Veh). Right: Quantification of NF-κB (**C**) and NFATc1 (**E**) nuclear translocation analyzed in the images obtained by confocal microscopy using ImageJ/Fiji software and expressed as MFI of nucleus/cytoplasm ratio quantified in 20 cells across 8 independent HPFs; original magnification, ×600 (*n* = 20). Scale bars: 5 μm. (**D** and **F**) NF-κB (**D**) or NFATc1 (**F**) nuclear translocation by ELISA-based assay. Each circle represents an independent culture (*n* = 3). (**G**–**J**) Quantification in COCA cells treated as in **C** of pro–IL-1β and TNF-α mRNA by qRT-PCR (**G** and **H**) and pro–IL-1β and TNF-α proteins in cell extracts (**I**) or culture supernatants (**J**). In **B**, **D**, and **F**–**J**, each circle represents 1 cell culture (**B**, **D**, and **G**–**I**, *n* = 3; **F** and **J**, *n* = 4). Means ± 1 SD were analyzed by 1-way ANOVA and post hoc Student-Newman-Keuls test; *P < 0.05, **P < 0.01, ***P < 0.001, ****P < 0.0001.

**Figure 6:**
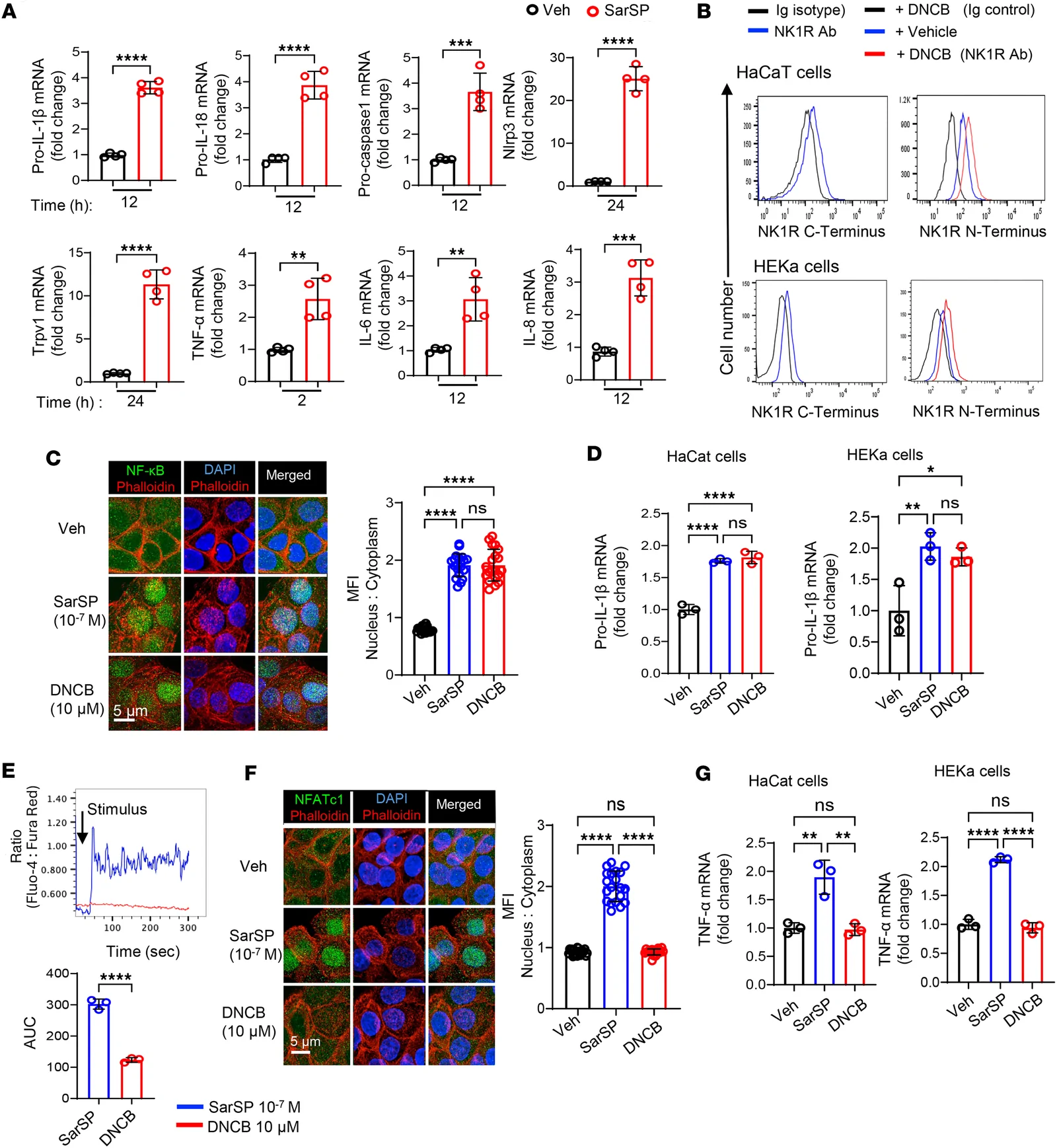
SP-NK1R signaling of human keratinocytes increases transcripts encoding proteins of the NLRP3 inflammasome and proinflammatory cytokines. (**A**) Quantification (by qRT-PCR) of transcripts for proteins of the NLRP3 inflammasome and proinflammatory cytokines in HaCaT cells treated with SarSP (10^–7^ M) at indicated time points. Each circle represents 1 keratinocyte culture (*n* = 4). (**B**) Left: Expression (by FACS) of functional NK1R assessed by the presence of the C-terminus in HaCaT and HEKa cells. Right: Expression of the N-terminus of NK1R in HaCaT and HEKa cells treated for 2 hours with DNCB (10 μM) or vehicle (Veh). One experiment of 3 is shown. (**C** and **F**) Left: Representative images by confocal microscopy of NF-κB (**C**) or NFATc1 (**F**) nuclear translocation in HaCaT cells treated with SarSP, DNCB, or DNCB vehicle. Right: Quantification of NF-κB (**C**) or NFATc1 (**F**) nuclear translocation analyzed with ImageJ/Fiji in images obtained by confocal microscopy, expressed as MFI of nucleus/cytoplasm ratio quantified in 20 cells across 8 independent HPFs; original magnification, ×600 (*n* = 20). Scale bars: 5 μm. (**D** and **G**) Quantification by qRT-PCR of transcripts for pro–IL-1β (**D**) and TNF-α (**G**) in HaCaT and HEKa cells treated with SarSP (10^–7^ M), DNCB (10 μM), or vehicle. Each circle represents 1 cell culture (*n* = 3). (**E**) Analysis of Ca^2+^ flux (FACS) induced by SarSP or DNCB in HaCaT cells. Top: One representative experiment of 3 is shown. Bottom: Comparison of Ca^2+^ flux by calculation of the area under the curve (AUC) (with FlowJo). Each circle represents 1 cell culture (*n* = 3). Means ± 1 SD were analyzed by 1-way ANOVA with post hoc Student-Newman-Keuls test; *P < 0.05, **P < 0.01, ***P < 0.001, ****P < 0.0001.

**Figure 7:**
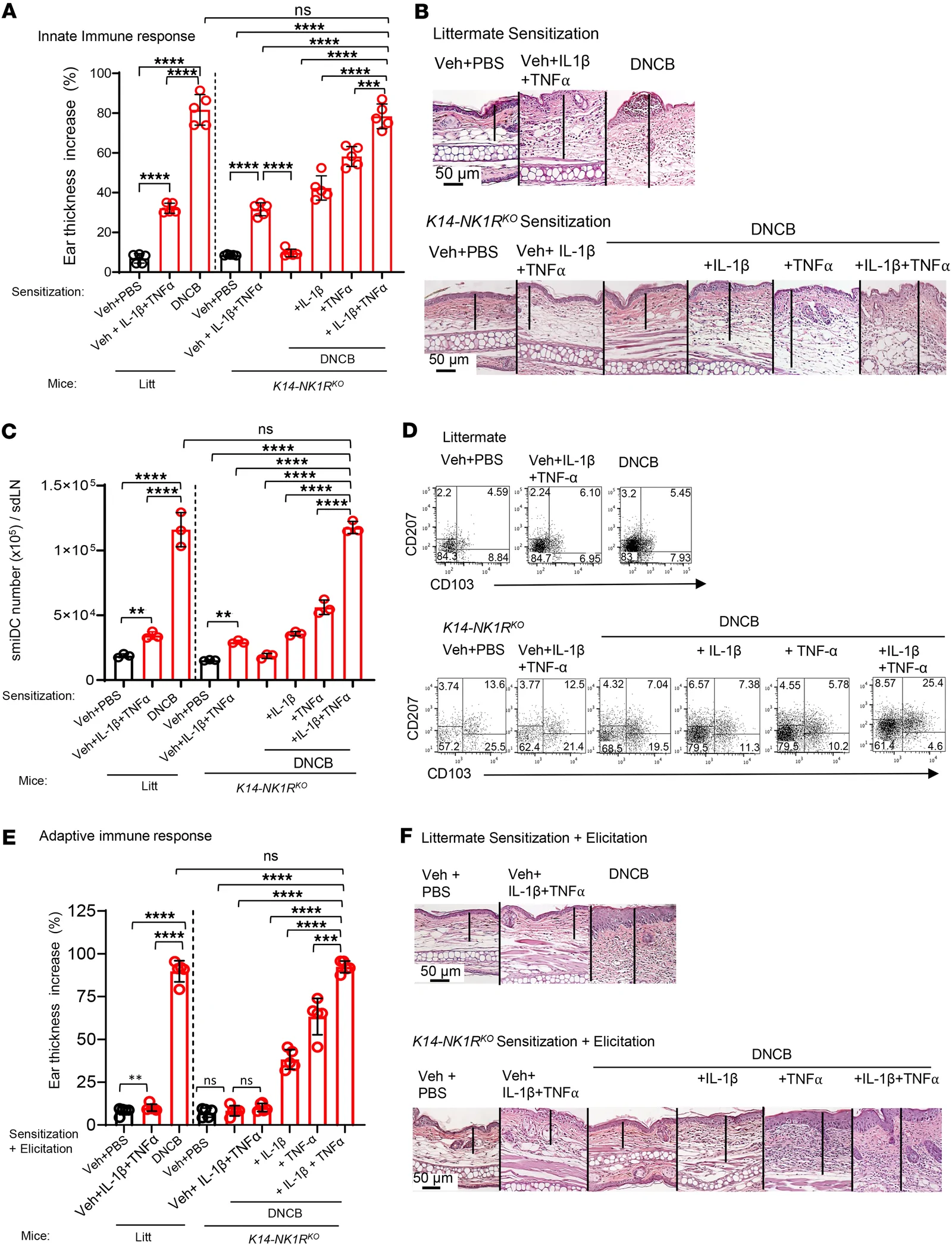
TNF-α and IL-1β restore the innate and adaptive immune response of DNCB CHS in *K14^Cre/+^ NK1R^KO^* mice. (**A**) Innate immune response by percentage of ear thickness 24 hours after sensitization of *K14^Cre/+^ NK1R^KO^* (*K14-NK1R^KO^*) mice treated on the right ear with topical DNCB alone or with IL-1β, TNF-α, or both (i.d.). *Cre^neg^* littermates (Litt) were treated with topical DNCB alone. Controls included mice treated with topical vehicle (Veh) plus PBS (i.d.), or topical vehicle and IL-1β plus TNF-α (i.d.). Each circle represents 1 mouse (*n* = 5). (**B**) Microscopic images of mouse ears shown in **A** illustrating epidermal-dermal thickness (vertical lines) and skin-infiltrating leukocytes. Scale bars: 50 μm. (**C** and **D**) Quantification (FACS) of skin-migratory dendritic cells (smiDCs) in skin-draining lymph nodes (sdLNs) 72 hours after sensitization as in **A**. Each circle represents smiDC number per individual sdLN (*n* = 3). (**D**) Representative dot plots of the smiDCs in sdLNs. (**E**) Effector immune response by percentage of ear thickness in *K14-NK1R^KO^* mice sensitized in the abdominal skin with DNCB alone or with IL-1β, TNF-α, or both (i.d.) and elicited in the right ear 5 days later with topical DNCB alone or with IL-1β, TNF-α, or both (i.d.). *Cre^neg^* littermates were sensitized and elicited with DNCB alone. Controls included mice sensitized and elicited with topical vehicle plus PBS (i.d.), or topical vehicle and IL-1β plus TNF-α (i.d.). Each circle represents 1 mouse (*n* = 5). (**F**) Microscopic images of the ears of mice in **E** 48 hours after elicitation, showing epidermal-dermal thickness (vertical lines) and skin-infiltrating leukocytes. Scale bars: 50 μm. (**B** and **F**) H&E; original magnification, ×200. Means ± 1 SD were analyzed by 1-way ANOVA and post hoc Student-Newman-Keuls test; **P < 0.01, ***P < 0.001, ****P < 0.0001.

## Version 1 09/01/2026

In-Press Preview

## Version 2 10/08/2026

Electronic publication
