# Supplementary material for: Substance P–NK1R signaling of keratinocytes initiates the skin inflammatory environment required for allergic contact dermatitis
Source: JCI Insight. 2026 Sep 1;11(19):e205826. doi: 10.1172/jci.insight.205826 (PMC13726421; doi:10.1172/jci.insight.205826)
Supplement: Supplementary file 1 [file jciinsight-11-205826-s251.pdf]

Supplemental Material for

Substance P-NK1R signaling of keratinocytes initiates the skin inflammatory environment required for allergic contact dermatitis

Sumeet Manandhar[1], Mohna Bandyopadhyay[1], Olga A. Tkacheva[1], William J. Shufesky[2,3], Gregory A. Gibson[4], Simon C. Watkins[4,5,6], Adrian E. Morelli[2,3,5] and Adriana T. Larregina,[1,5,6]

[1]Department of Dermatology, University of Pittsburgh, School of Medicine, Pittsburgh, PA, USA. [2]Thomas E. Starzl Transplantation Institute, University of Pittsburgh, School of Medicine, Pittsburgh, PA, USA. [3]Department of Surgery, University of Pittsburgh, School of Medicine, Pittsburgh, PA, USA. [4]Department of Cell Biology and Center for Biological Imaging, University of Pittsburgh, School of Medicine, Pittsburgh, PA, USA. [5]Department of Immunology, University of Pittsburgh, School of Medicine. [6]The McGowan Center for Regenerative Medicine, University of Pittsburgh, Pittsburgh, PA, USA.

The PDF includes Supplemental Table 1 and Supplemental figures S1-S5

Supplemental Table 1: List of antibodies used in the present study specifying name, clone, provider and catalog number

Supplemental Figure S1: Substance P and the NK1R are required for the skin irritant effects of DNCB and croton oil.

Supplemental Figure S2: Substance P synthesis and NK1R expression in keratinocytes are required for efficient effector CHS.

Supplemental Figure S3: Stimulation of keratinocytes with SP or SarSP increases transcripts of proteins involved in the NLRP3 inflammasome and of TNF-α.

Supplemental Figure S4: SarSP induces pro-IL-1β and TNF-α transcripts in HaCaT cells in a dose dependent manner and promotes intracellular Ca^{2+} increase by signaling via the NK1R.

Supplemental Figure S5: TNF-α and IL-1β restore the innate and adaptive immune responses of DNCB CHS in *K14$^{Cre/+}$NK1R^{KO}* mice.

Supplemental Table 1: List of antibodies used in the present study specifying name, clone, provider and catalog number

ANTIBODY	SOURCE	CATALOG #
Anti-mouse CD8α (Clone 53-6.7) PacBlue	BioLegend	Cat # 100725
Anti-mouse CD11b (Clone M1/70) PerCP-Cy5	BD	Cat # 550993
Anti-mouse CD11c (Clone N418), BV510	BD	Cat # 562949
Anti-mouse CD45 (Clone 30-F11), PE	BD	Cat # 553081
Anti- mouse CD103 (Clone M290) BUV395	BD	Cat # 740238
Anti-mouse/human CD207 (Clone 4C7), APC	BioLegend	Cat # 144205
Anti-mouse CD301b (Clone URA-1)	BioLegend	Cat # 146809
Anti-mouse I-Ab (clone AF120.1) APC-Cy7	BioLegend	Cat # 116420
Anti-human NK1R N-terminus, APC (Clone 694501)	RD	Cat # FAB 66871A
Anti-mouse / rat / human NK1R extracellular domain (rabbit polyclonal), ATTO488	Alomone Labs	Cat # ATR-001-AG
Anti-mouse / human NK1R C-terminus, PE (Clone D-11)	Santa Cruz	Cat # SC-365091
Anti-mouse / human NF-κBp65 (Clone J053B5)	BioLegend	Cat # 662602
Anti-mouse / human NFATc1 (Clone 7A6)	BioLegend	Cat # 649602
Fluo-4AM	Invitrogen	Cat # 14201
Fura Red™ AM Cell Permeant	Invitrogen	Cat # F3021
DAPI	Thermo Fisher	Cat # D1306
Fixable Viability Dye eFluor 780	eBioscience	Cat # 65-0865-14
CountBright™ Absolute Counting Beads	Invitrogen	Cat # C36950
AF647- Phalloidin	Invitrogen	Cat # A22287
Goat anti-mouse IgG-AF488 (polyclonal)	Jackson ImmunoResearch INC	Cat # 115-545-003
Goat anti-rabbit IgG AF488 (polyclonal)	Jackson ImmunoResearch INC	Cat # 111-545-003

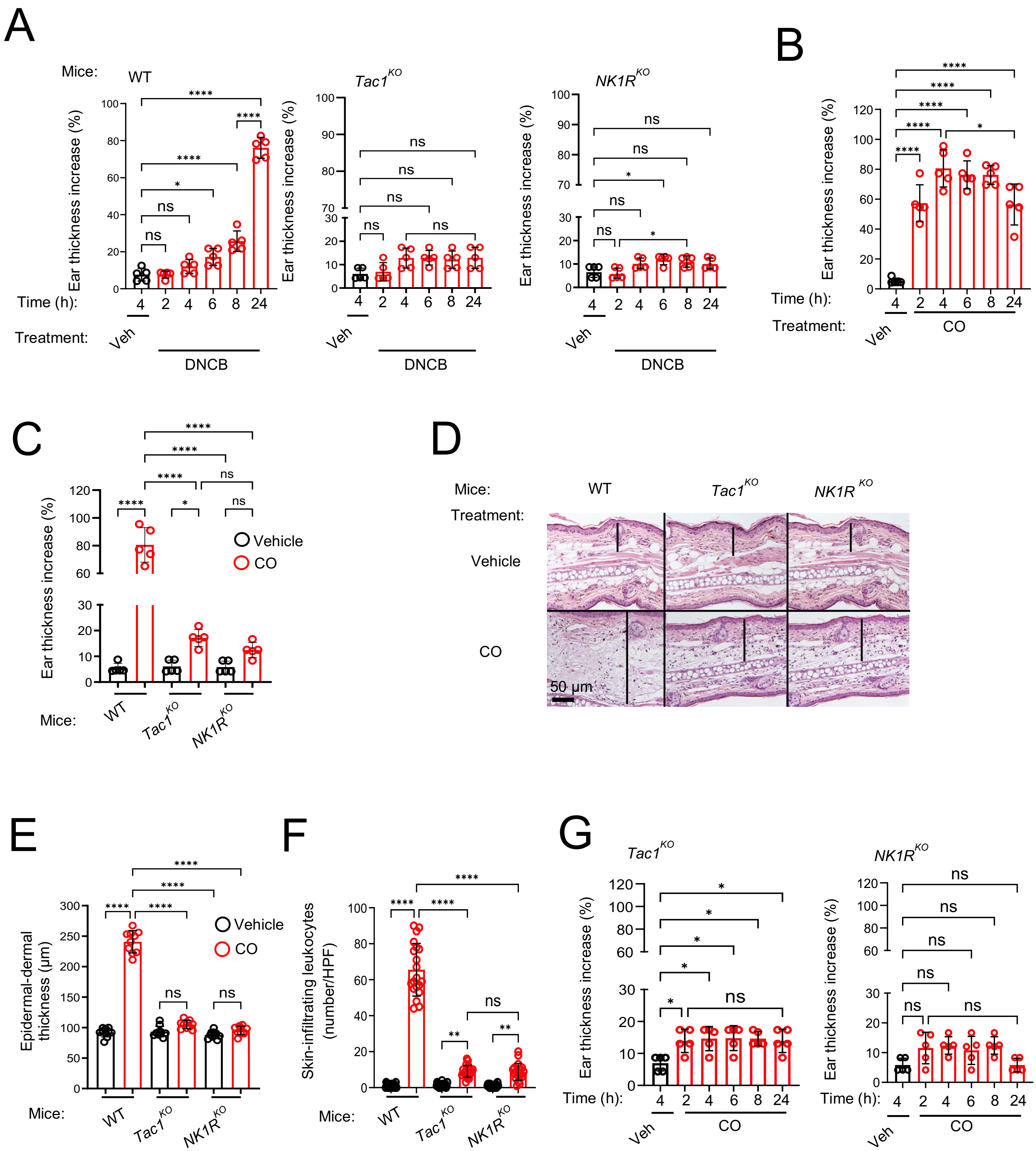

Supplemental Figure S1

Figure S1: Substance P and the NK1R are required for the skin irritant effects of DNCB and croton oil. (**A**) Percent of ear thickness increase in WT, *Tac1*^{KO} (without SP) or *NK1R*^{KO} mice at the indicated time points following skin topical application of DNCB. (**B**) Percent of ear thickness increase in WT mice at the indicated time points following skin topical application of croton oil (CO) or vehicle (Veh). (**C**) Percent of ear thickness increase in WT, *Tac1*^{KO} and *NK1R*^{KO} mice 4 h after skin topical application of CO. (**D**) Microscopic images of the ears of mice in (**C**) showing epidermal-dermal thickness (vertical lines), edema, and skin-infiltrating polymorphonuclear leukocytes, H&E 200x. (**E** and **F**) Quantification of epidermal-dermal thickness (**E**) and infiltrating leukocytes (**F**) in skin samples shown in (**D**). In (**E**) each circle represents one measurement (n=10). In (**F**) each circle represents the number of leukocytes per microscopic HPF, 500x, (n=20). (**G**) Percent of ear thickness in *Tac1*^{KO} and *NK1R*^{KO} after skin topical application CO at the indicated time points. (**A, B, C** and **G**), each circle represents 1 mouse, (n=5). (**A**-**C** and **E-G)** Means ± 1 SD were analyzed by 1-way ANOVA and post-hoc Student-Newman-Keuls test. ns: not significant, *p<0.05, ****p<0.0001.

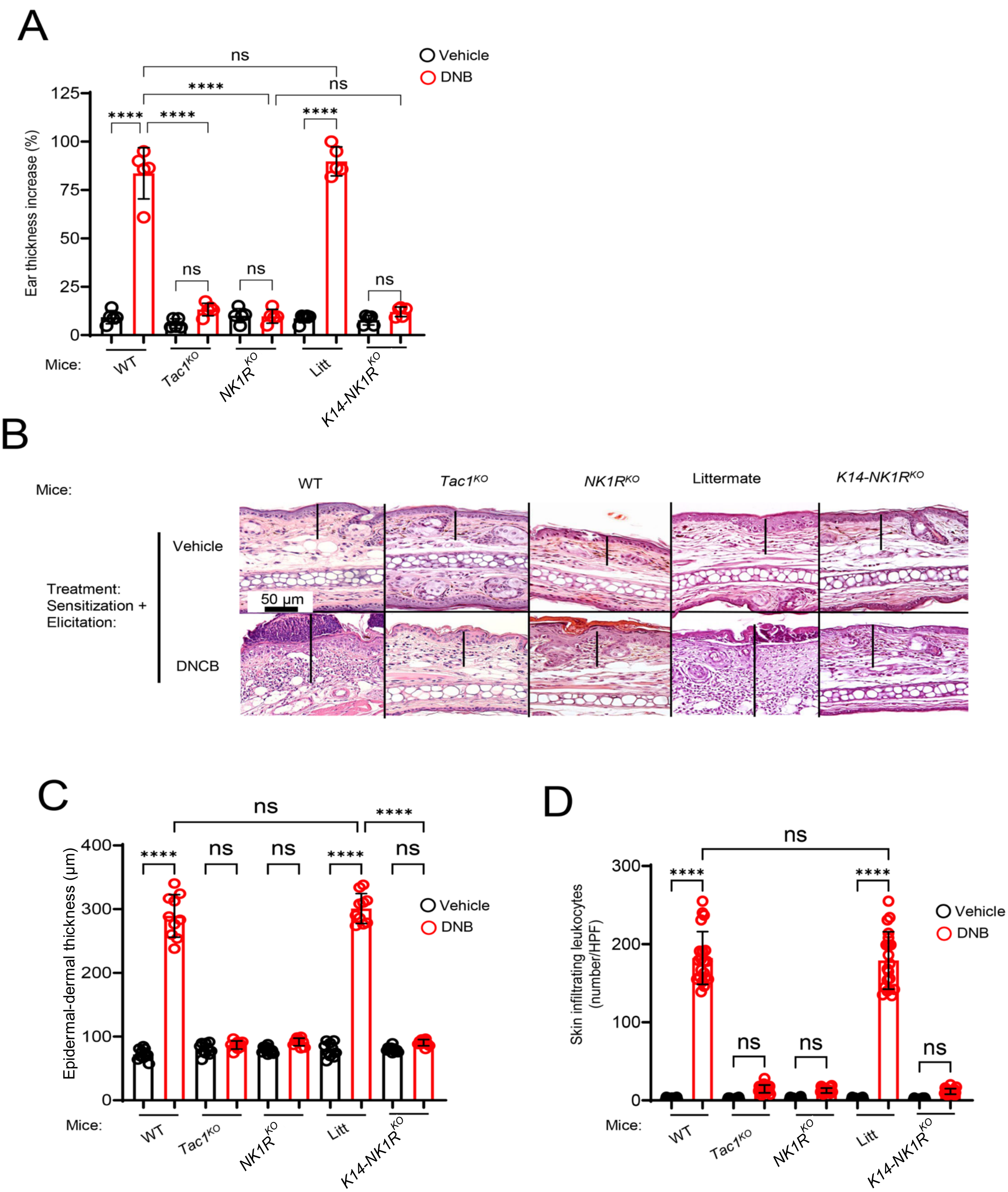

Supplemental Figure S2

Figure S2: Substance P synthesis and NK1R expression in keratinocytes are required for efficient effector CHS. (**A-D**) Skin effector immune response in WT, global *Tac1*^{KO} (without SP), global *NK1R*^{KO} and *K14*$^{Cre/+}$*NK1R*^{KO} (*K14-NK1R*^{KO}) (with specific deletion of NK1R in keratinocytes) and their littermate control mice, induced by DNCB sensitization on the lower abdominal skin followed by elicitation 5 d later, on the right ear. (**A**) Percent of ear thickness increase assessed 48 h after CHS elicitation. Each circle represents one mouse, (n=5). (**B**) Representative microscopic images of the ears of mice in (**A**), showing epidermal-dermal thickness (vertical lines) and skin-infiltrating leukocytes. H&E, 200x. (**C**) Quantification of epidermal-dermal thickness in ear samples of mice in (**A)**. Each circle represents one measurement (n=10). (**D**) Quantification of skin-infiltrating leukocytes in the ear samples of mice in (**A**). Each circle represents the number of leukocytes per microscopic HPF, 500x (n=20). Means ± 1 SD were analyzed by 1-way ANOVA and post-hoc Student-Newman-Keuls test; ns: not significant, ****p<0.0001.

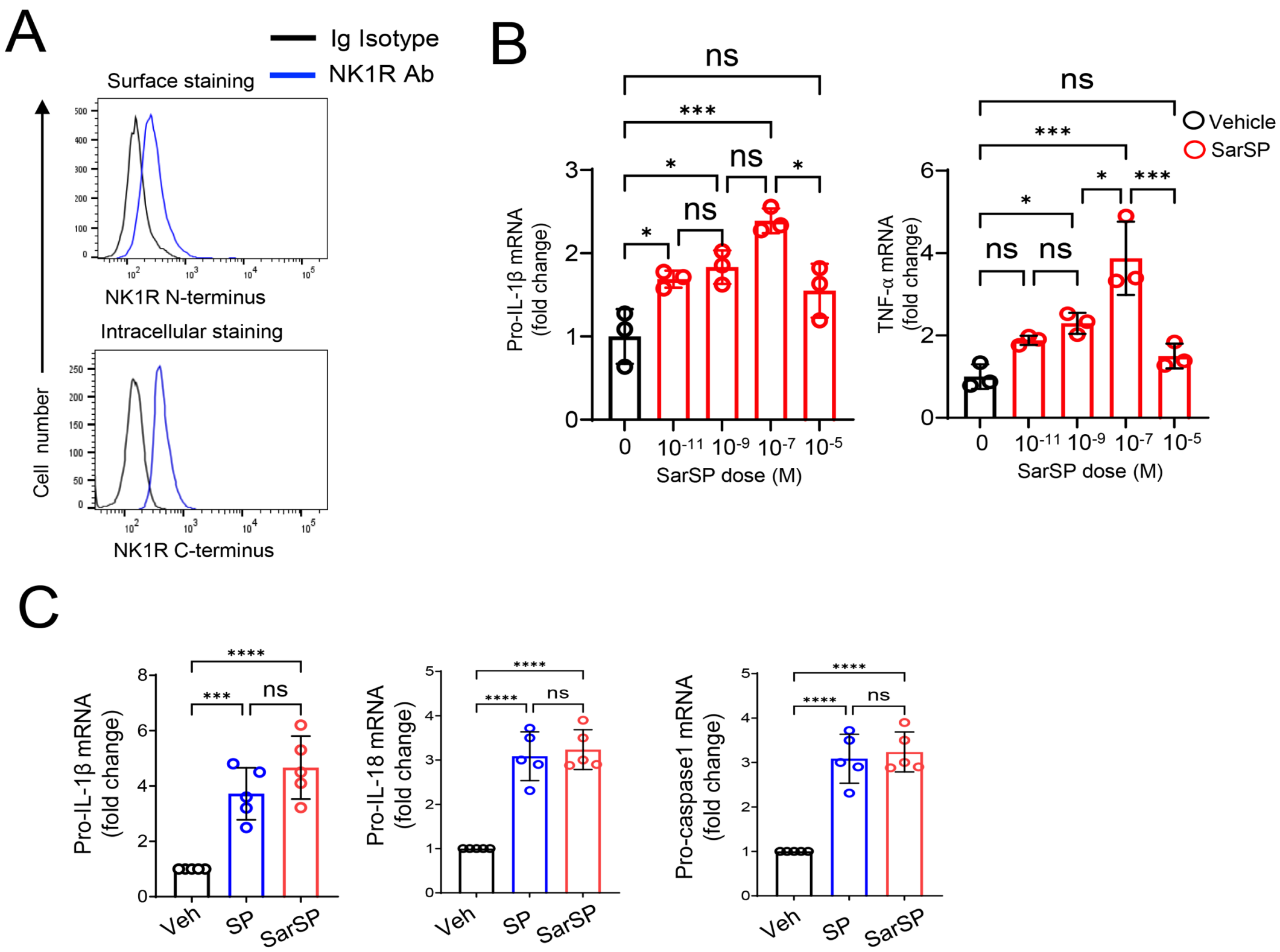

Figure S3: Stimulation of keratinocytes with SP or SarSP increases transcripts of proteins involved in the NLRP3 inflammasome and of TNF-α. (**A**) Constitutive expression of the NK1R in COCA cells labelled with Ab recognizing the N-terminus or the C-terminus of the NK1R and analyzed by FACS. (**B**) Increase of IL-1β and TNF-α transcripts in COCA cells treated with the indicated doses of SarSP. (**C**) Comparative analysis of the effects of SP and SarSP (both at a concentration of 10^{-7} M) in the expression pro-IL-1β, pro-IL-18 and pro-caspase1 transcripts in COCA cells. In (**B** and **C**), each circle represents one cell culture, (**B**), n=3, (**C**), n=5. (**B** and **C**) Means ± 1 SD were analyzed by 1-way ANOVA and post-hoc Student-Newman-Keuls test; ns: not significant, *p<0.05, ***p<0.001, ****p<0.0001.

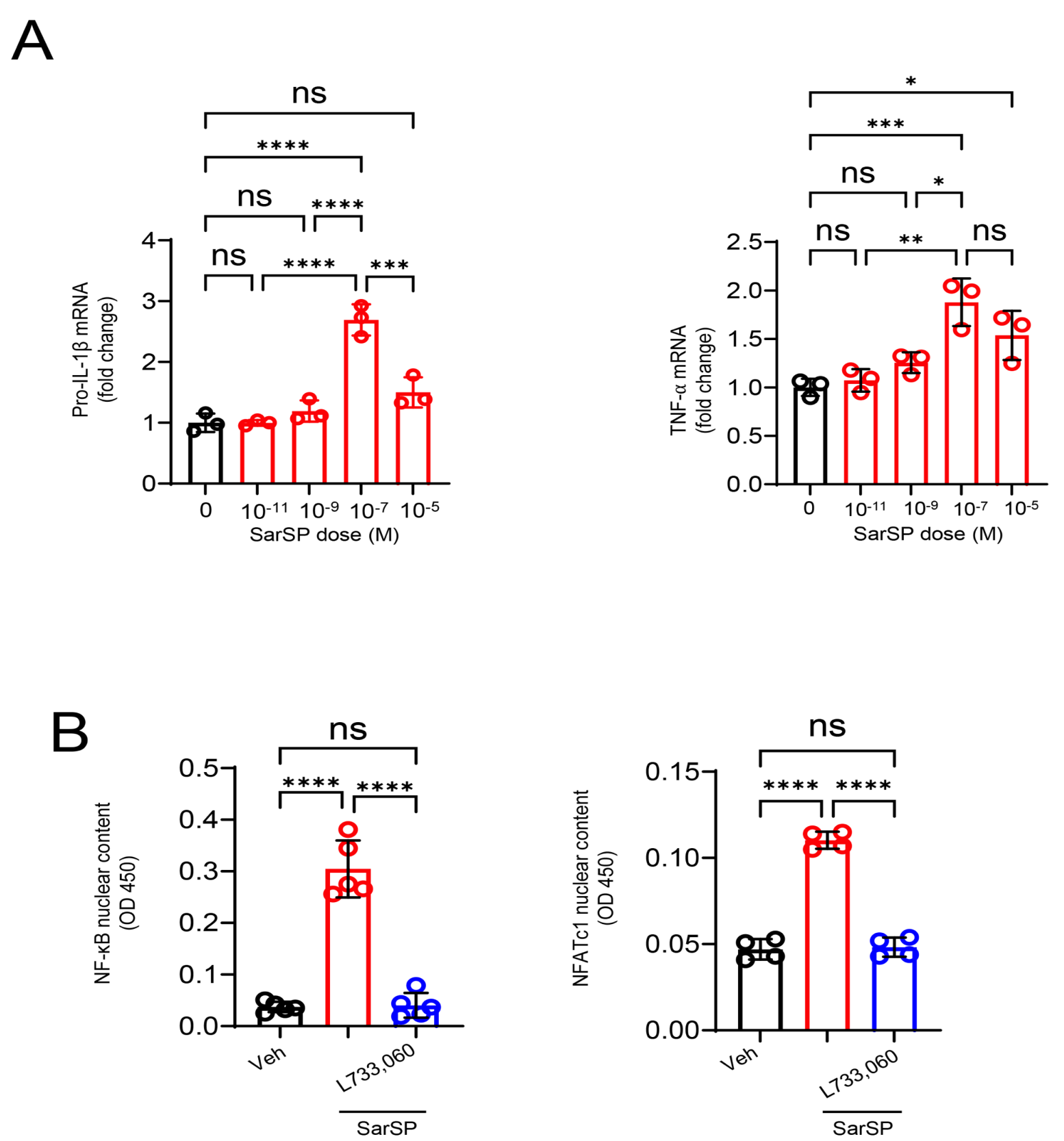

Figure S4: SarSP induces pro-IL-1β and TNF-α transcripts in HaCaT cells in a dose dependent manner and promotes NF-κB and NFATc1 nuclear translocation by signaling via the NK1R. (**A**) Increase of pro-IL-1β and TNF-α transcripts assessed by qRT-PCR in HaCaT cells treated with the indicated doses of SarSP. (**B**) Nuclear translocation of NF-κB and NFATc1 induced by SarSP in HaCaT cells in the presence or not of the human specific NK1R antagonist L733,060. In (**A** and **B**) each circle represents one cell culture, (**A**) n=3, (**B** left) n=5, (**B** right) n=4. Means ± 1 SD were analyzed by 1-way ANOVA and post-hoc Student-Newman-Keuls test; ns: not significant, *p<0.05, **p<0.01, ***p<0.001, ****p<0.0001.

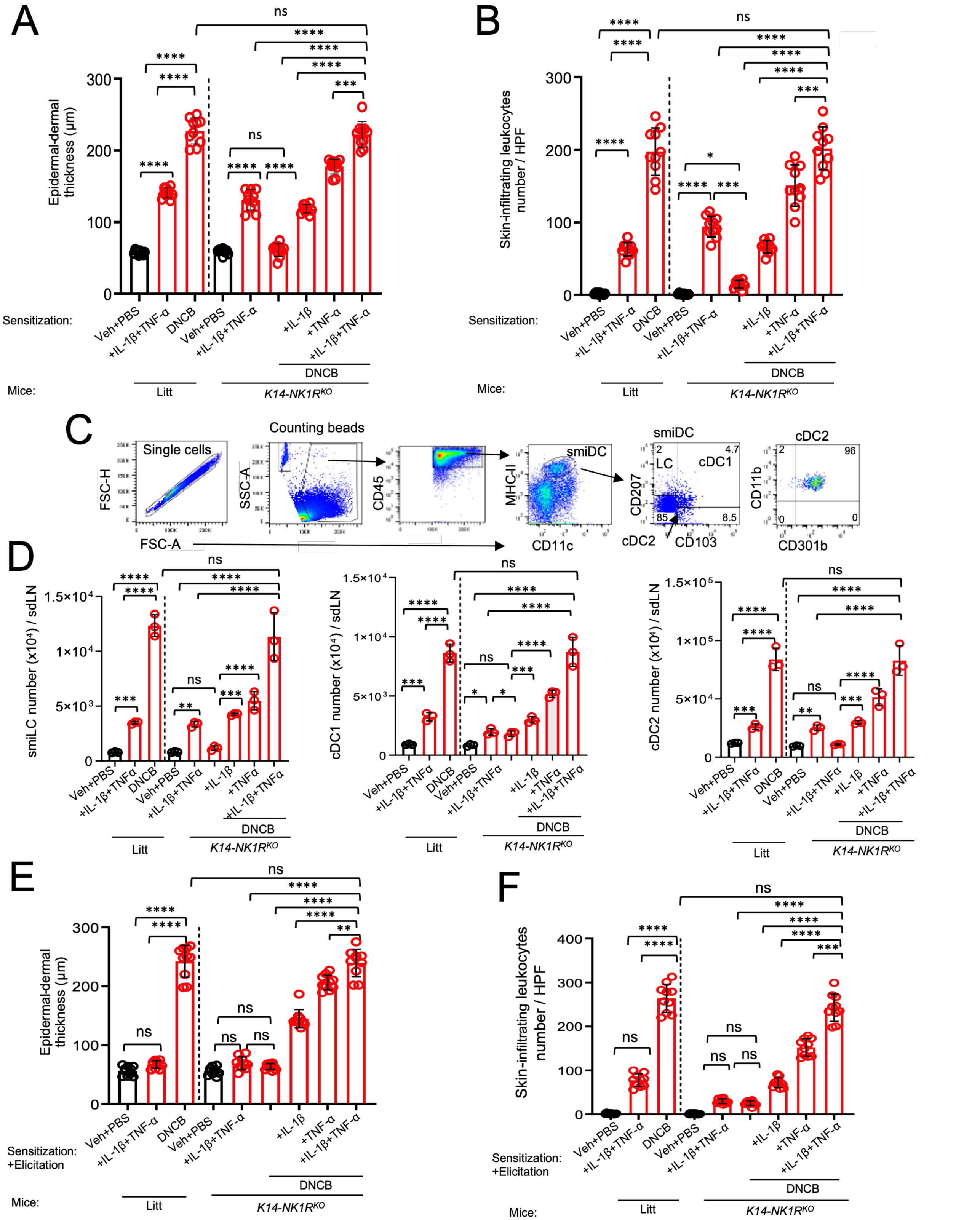

Supplemental Figure S5

Figure S5: TNF-α and IL-1β restore the innate and adaptive immune responses of DNCB CHS in *K14^Cre/+^NK1R^KO^* mice. (**A**) Quantification of epidermal-dermal thickness 24 h after sensitization of *$K14^{Cre/+}NK1R^{KO}$* (*$K14\text{-}NK1R^{KO}$*) mice treated on the right ear with topical DNCB alone or with IL-1β, TNF-α, or both (i.d.). *Cre^{Neg}* littermates (Litt) were treated with topical DNCB alone. Controls included mice treated with topical DNCB-vehicle (veh) plus PBS (i.d.), or topical veh and IL-1β plus TNF-α (i.d.). Each circle represents one measurement, 200x, (n=10). (**B**) Quantification of infiltrating-leukocytes in the skin of mice in (**A**). Each circle represents the quantification of cells per HPF, 500x, (n=10). (**C**) Gating strategy (by FACS) used to identify the subsets of skin-migratory dendritic cell (smiDC) in skin-draining lymph nodes (sdLN) of mice. (**D**) Quantification of smiDC subsets in the sdLN of mice 72 h after sensitization as described in (**A**). Each circle represents smiDC number per individual sdLN (n=3). (**E**) Quantification of epidermal-dermal thickness 48 h after elicitation in (*$K14\text{-}NK1R^{KO}$*) mice sensitized on the abdominal skin and elicited 5 d later on the right ear. Sensitization and elicitation were performed by topical application of DNCB alone or with IL-1β, TNF-α, or both (i.d.). *Cre^{Neg}* littermates (Litt) were sensitized and elicited with topical DNCB alone. Controls included mice treated topically with topical veh plus PBS (i.d), or topical veh and IL-1β plus TNF-α (i.d). Each circle represents one measurement, 200x, (n=10). (**F**) Quantification of infiltrating-leukocytes in the skin of mice treated as in (**E**). Each circle represents the number of cells per HPF, 500x, (n=10). Means ± 1 SD were analyzed by 1-way ANOVA and post-hoc Student-Newman-Keuls test; ns: not significant, *$p<0.05$, **$p<0.01$, ***$p<0.001$, ****$p<0.0001$.
